# NextDenovo: an efficient error correction and accurate assembly tool for noisy long reads

**DOI:** 10.1186/s13059-024-03252-4

**Published:** 2024-04-26

**Authors:** Jiang Hu, Zhuo Wang, Zongyi Sun, Benxia Hu, Adeola Oluwakemi Ayoola, Fan Liang, Jingjing Li, José R. Sandoval, David N. Cooper, Kai Ye, Jue Ruan, Chuan-Le Xiao, Depeng Wang, Dong-Dong Wu, Sheng Wang

**Affiliations:** 1 GrandOmics Biosciences, Beijing, 102206 China; 2 Key Laboratory of Genetic Evolution and Animal Models, Kunming Institute of Zoology, Chinese Academy of Sciences, Kunming, 650223 China; 3 Centro de Investigación de Genética y Biología Molecular (CIGBM), Instituto de Investigación, Facultad de Medicina, Universidad de San Martín de Porres, Lima, 15102 Peru; 4 Institute of Medical Genetics, Cardiff University, Heath Park, Cardiff, CF14 4XN UK; 5 School of Automation Science and Engineering, Faculty of Electronic and Information Engineering, Xi’an Jiaotong University, Xi’an, China; 6 Shenzhen Branch, Guangdong Laboratory of Lingnan Modern Agriculture, Genome Analysis Laboratory of the Ministry of Agriculture and Rural Affairs, Agricultural Genomics Institute at Shenzhen, Chinese Academy of Agricultural Sciences, Shenzhen, 518120 China; 7 State Key Laboratory of Ophthalmology, Zhongshan Ophthalmic Center, Sun Yat-Sen University, #7 Jinsui Road, Tianhe District, Guangzhou, China; 8 Kunming Primate Research Center, and National Research Facility for Phenotypic and Genetic Analysis of Model Animals (Primate Facility), National Resource Center for Non-Human Primates, Kunming Institute of Zoology, Chinese Academy of Sciences, Kunming, 650107 China; 9 Yunnan Key Laboratory of Biodiversity Information, Kunming Institute of Zoology, Chinese Academy of Sciences, Kunming, China; 10 Kunming Natural History Museum of Zoology, Kunming Institute of Zoology, Chinese Academy of Sciences, Kunming, China

**Keywords:** Long reads, Genome assembly, Error-correction, Human genomes, Segmental duplication

## Abstract

Long-read sequencing data, particularly those derived from the Oxford Nanopore sequencing platform, tend to exhibit high error rates. Here, we present NextDenovo, an efficient error correction and assembly tool for noisy long reads, which achieves a high level of accuracy in genome assembly. We apply NextDenovo to assemble 35 diverse human genomes from around the world using Nanopore long-read data. These genomes allow us to identify the landscape of segmental duplication and gene copy number variation in modern human populations. The use of NextDenovo should pave the way for population-scale long-read assembly using Nanopore long-read data.

**Supplementary Information:**

The online version contains supplementary material available at 10.1186/s13059-024-03252-4.

## Background

An accurate and complete genome is a prerequisite for studying the evolution of species. Third-generation long-read sequencing platforms, such as PacBio single-molecule real-time (SMRT) [1] and Oxford Nanopore (ONT) [2], promise to overcome the challenges that are inherent to short-read sequencing and have the potential to resolve most complex and repetitive genomic regions. To this end, they have become the mainstream method of sequencing for genome assembly. The high-fidelity (HiFi) reads recently produced by PacBio display superior performance to de novo assembly [3–5]. However, they generally have an average length of ~ 15 kilobases (kb) and hence are unable to span long tandem or highly homologous multi-copy repeats, which occur widely throughout large genomes, but very specifically in some regions such as centromeres [3, 6]. ONT sequencing can generate > 100-kb “ultra-long” reads, which can be used to fill the final gaps of an assembly, most of which are located in these regions [7, 8]. This approach was first used successfully in the assembly of a human centromere (chromosome Y) [9] and an entire chromosome (chromosome X) [10] and was then combined with HiFi data to assemble a complete human genome [8]. Despite these successes, a single linear reference genome is insufficient to represent the entire genome sequence of a species, and there is an urgent need to construct pan-genomes for population genome studies [11–13]. ONT sequencing is characterized by lower cost, higher throughput, and a faster turnaround time than PacBio HiFi sequencing, and since it requires less genomic DNA, it can be used anywhere for sampling and sequencing by portable devices. It is therefore ideally suited for pan-genome projects, especially those with limited budgets or urgent deadlines.

For genome assembly from noisy long ONT reads, two commonly used strategies have been employed, viz. “correction then assembly” (CTA, an assembler first corrects errors in the reads and then uses the corrected reads for assembly) and “assembly then correction” (ATC, an assembler uses error-prone reads to assemble the genome and then corrects errors in the assembled genome); the former (such as Necat [14] and Canu [15]) is usually slower than the latter (such as Wtdbg2 [16] and Flye [17]), because read-level error correction requires much more computational resources than contig-level polishing (a step to correct errors in the assembly). However, in terms of the assembly of segmental duplications/repeats, and especially for large plant genome assemblies, the CTA-based strategy usually has an enhanced ability to distinguish different gene copies and produce more accurate and continuous assemblies [14, 15, 18].

Here, we present NextDenovo, a highly efficient error correction and CTA-based assembly tool for noisy long reads. We first provide an overview of the NextDenovo pipeline and then compare it to other error correction and assembly tools using four non-human genomes and 35 human genomes. We show that NextDenovo represents an optimal choice for error correction and genome assembly when working with noisy long reads, especially for large repeat-rich genomes.

## Results

### Overview of the NextDenovo pipeline

**Fig. 1:**
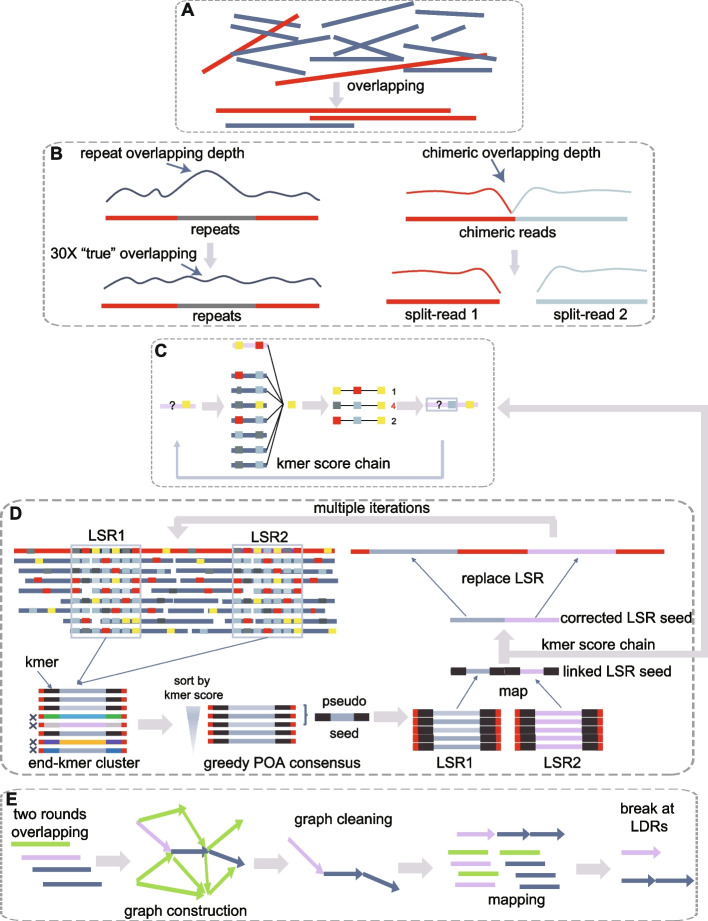
NextDenovo pipeline. **A** Overlapping reads. **B** Alignments erroneously caused by repeats were filtered out and chimeric reads were split. **C** A confidence score was calculated for a given allele at each position with a fixed 3-mer, and the allele with the maximum score was selected as the correct base. The colored rectangles represent the different bases. **D** NextDenovo first identifies all LSRs at the raw reads, extracts each subsequence spanning these LSRs, and assigns a kmer score to each subsequence. Subsequently, NextDenovo filters out the subsequences with lower scores and produces a pseudo-LSR seed using a greedy POA consensus algorithm, all pseudo-LSR seeds from the same seed being linked as the reference, and all subsequences being mapped to this reference while the KSC algorithm is reapplied to produce a corrected pseudo seed. Then, the corrected LSRs are inserted into the corresponding positions in the raw reads to generate the final corrected reads. **E** NextDenovo calculates dovetail alignments by two rounds of overlapping, constructs an assembly graph, removes transitive edges, tips, bubbles, and edges with low scores, and generates contigs. Finally, NextDenovo maps all seeds to contigs and breaks a contig if it possesses low-quality regions

As with other CTA assemblers, NextDenovo first detects the overlapping reads (Fig. 1A), then filters out the alignments caused by repeats, and finally splits the chimeric seeds based on the overlapping depth (Fig. 1B). NextDenovo employs the Kmer score chain (KSC) algorithm which was used by our previously published polisher tool, NextPolish [19], to perform the initial rough correction (Fig. 1C). Repeated regions typically contain numerous noisy or incorrect overlap alignments. These regions are usually characterized by lower accuracy after the initial correction, but they are nonetheless important for distinguishing different duplicates during the subsequent graph cleaning procedure. Therefore, NextDenovo used a heuristic algorithm to detect these low-score regions (LSRs) during the traceback procedure within the KSC algorithm. For the LSRs, a more accurate algorithm, derived by combining the partial order alignment (POA) [20] and KSC, was used. In detail, each subsequence spanning an LSR was collected, and a kmer set at the flanking sequences of this LSR was generated. Then, each subsequence was assigned a matched kmer score based on this kmer set. Subsequences with a lower kmer score (mainly caused by heterozygosity or repeats) were filtered out. The six longest subsequences ranked by kmer score were used to produce a pseudo-LSR seed by a greedy POA consensus algorithm. All pseudo-LSR seeds from the same seed were linked as the reference, all subsequences from this seed were mapped to this reference, and the KSC algorithm was applied again to produce a corrected pseudo seed. This procedure was called multiple times to improve the accuracy of the LSRs. Finally, each LSR was extracted from the corrected pseudo seed and inserted into the corresponding position of the primary corrected seed as the final corrected seeds (Fig. 1D).

The corrected seeds were subjected to two rounds of pairwise overlapping to identify dovetail alignments (two reads overlapping each other in an end-to-end fashion). The first round used an efficacious parameter set designed to rapidly detect candidate dovetail alignments, which usually contain incorrect alignments or imprecise alignment boundaries. Thus, for these candidate dovetail alignments, a rigorous parameter set was used to produce more accurate alignments. Next, a directed string graph was constructed and transitive edges were removed as with most existing assemblers (if there are edges from A to B, B to C, and A to C, then the edge from A to C is removed from the graph as it can be inferred from the edges between A to B and B to C). We used the “best overlap graph” (BOG) algorithm to remove edges for non-repeat nodes (repeat nodes were defined as nodes with indegree or outdegree larger than a threshold). For repeated nodes, we found that the BOG algorithm typically removes the corrected edges and breaks the graph connectivity. To fix this problem, we only removed a repeat edge if its alignment identity, length, and transitive score (see the “ Methods” section) were less than their corresponding thresholds. Subsequently, the tips were removed and the bubbles were resolved. Finally, the graph usually contained some linear paths (no branches and repeated nodes) connecting some complex subgraphs that contained many repeat nodes. We used a greedy progressive graph cleaning strategy to simplify these complex subgraphs, that is, a series of increasingly stringent thresholds were used to filter edges while maintaining connectivity between incoming and outgoing nodes. Finally, all paths were broken at the node connected with multi-paths, and the contigs were output from these broken linear paths. To further reduce the possibility of misassemblies, we mapped all seeds to the contigs and broke a contig at the connection point between two nodes if it had a lower mapping depth region (LDR) (Fig. 1E).

### Benchmarking the error correction module

Error correction is a crucial step for the CTA assembler. Therefore, we benchmarked the error correction performance (including correction speed, corrected data size, error rate and chimeric reads rate of corrected reads) of NextDenovo against Consent (v2.2.2) [21], Canu (v2.0), and Necat (v0.0.1) using simulated data and real biological data based on chromosome one of the human CHM13 genome (Table 1, Additional file 1: Table S1) [8].

**Table 1:** Statistics of ONT read error correction

Source	Software	Corrected bases rate (%)	Average length (bp)	Max length (bp)	Reads with chimeric alignments (%)	Mapped with > = 99% coverage (%)	Mapped with > = 97% identity (%)	Average error rate (%)	Wall clock time (hour)
Simulation (chr1, 62X)	Raw reads	-	22,884	279,538	**0.03**	73.28	0.00	12.37	-
	Consent	84.72	**23,597**	**280,569**	**0.03**	97.01	99.77	**0.14**	7.29
	NextDenovo	83.47	23,515	188,302	**0.03**	**99.58**	**99.96**	0.20	**2.43**
	Necat	**85.12**	23,542	264,959	0.08	98.46	98.83	0.55	2.75
	Canu	81.26	23,369	279,129	0.41	98.03	96.58	2.02	18.08
CHM13 (chr1, 72X)	Raw reads	-	**91,209**	499,238	17.07	81.19	0.18	8.57	-
	Consent	**99.18**	90,701	503,237	19.22	80.72	83.11	1.37	17.43
	NextDenovo	97.13	90,981	505,469	**10.70**	**89.10**	**89.31**	**0.90**	**1.83**
	Necat	98.13	89,170	**506,817**	11.43	88.30	88.67	0.99	2.98
	Canu	92.59	84,270	502,469	13.44	85.13	80.64	2.21	126.72

Only the primary alignments defined by minimap2 of each read were used for evaluation. Corrected base rate is the ratio of the size of the corrected reads to the size of the raw reads to be corrected. Reads with chimeric alignments are defined as reads that have supplementary alignments. Average error rate only uses the reads that are mapped with ≥ 80% coverage. All the software was tested on the same computer with 32 CPUs and 252 GB RAM of memory. Best results for each metric are highlighted in bold

In terms of the correction speed, NextDenovo demonstrated impressive performance, being 3.00, 7.44, and 1.13 times faster on simulated data and 9.51, 69.25, and 1.63 times faster on real data compared to Consent, Canu, and Necat, respectively. It is essential to note that the differences between simulated data and real data are primarily attributed to the latter being comprised of ONT “ultra-long” reads, the reads to be corrected having an average length of 91.21 kb, 3.99 times longer than the simulated data.

We conducted additional tests by simulating reads of varying lengths and correcting them using NextDenovo, Canu, and Necat (we were not successful in running Consent on these datasets). Interestingly, our findings revealed that as the read length increases, the time required for correction also increases. However, NextDenovo and Necat demonstrated only slight increases, while Canu exhibited a significant increase in processing time (Additional file 1: Table S2). Regarding the corrected data size, NextDenovo corrected 2.21% and 4.54% more data than Canu, but 1.65% and 1.25% and 1.00% and 2.05% fewer data than Necat and Consent, on simulated data and actual biological data, respectively. Further examination unveiled that about 0.93% of simulated reads and 2.36% of real biological reads could be corrected by either Canu or Consent, but not by NextDenovo (Additional file 1: Fig. S1). It is imperative to highlight that within this subset of uncorrected reads, 99.98% of simulated reads and 99.08% of real biological reads cannot be completely mapped to the reference genome (≥ 80% coverage). This indicates that the majority of these uncorrected reads were of extremely low quality or chimeric and were filtered out by NextDenovo to prevent any adverse effects on the subsequent assembly graph construction. Importantly, NextDenovo achieves an average error rate of 1.82% and 1.31% lower than Canu and 0.35% and 0.09% lower than Necat on simulated and real biological data, respectively, while Consent is found to perform well on simulated data but poorly on real data. It is worth mentioning that the average accuracy of corrected reads by NextDenovo exceeded 99%, closely matching the accuracy of the PacBio HiFi reads, whereas they are much longer than HiFi reads. Furthermore, a consistent error rate within the corrected reads is essential for subsequent graph cleaning procedures, as read alignment identities can be used to distinguish ambiguous edges in the assembly graph, especially when these edges are from different duplicates. Compared to simulated data, we found that the ONT reads from the real biological data tend to have higher errors in certain regions that NextDenovo can identify as LSRs. Benefitting from the heuristic algorithm that correct the LSRs with multiple iterations, NextDenovo produced ~ 89.31% of the corrected reads that have an accuracy of ≥ 97%, while the comparable figures were only 80.64% for Canu, 83.11% for Consent, 88.67% for Necat, and 0.18% for the raw data. Chimeric reads usually hinder assembly graph construction, resulting in misconnections and incorrect assembly results. NextDenovo can detect these chimeric reads and can split them at the LSRs or filter them based on their length after splitting; 89.10% of the corrected reads can be mapped to reference with ≥ 99% coverage, compared to 85.13% for Canu and 80.72% for Consent, while the comparable figure for Necat was slightly lower (88.30%) than with NextDenovo. We also investigated how accurate this chimeric read splitting process is. The results showed that only 0.07% of reads were split by error, lower than the 4.83% for Canu but higher than the 0.002% for Consent (a mis-split read was defined as a read that can be completely mapped to the reference without correction, but was not included in the corrected result or the length was significantly shorter after correction). This result is consistent with NextDenovo exhibiting the fewest chimeric alignments for read correction.

In summary, NextDenovo is able to correct reads at a faster speed, and the corrected reads contain fewer errors and are characterized by a higher uniform error rate and fewer chimeric alignments.

### Assembly evaluation on non-human genomes

We first evaluated NextDenovo in the context of the assembly of four non-human genomes (*Arabidopsis thaliana*, *Drosophila melanogaster*, *Oryza sativa*, and *Zea mays*) with the most widely used assemblers, Necat (v0.0.1), Canu (v2.0), Flye (v2.8), and Wtdbg2 (v2.5) on ONT data (Additional file 1: Table S1) and then used QUAST (v5.2.0) [22] to evaluate all assemblies concerning completeness (assembly size, gene completeness), accuracy (number of misassemblies and Phred-scaled base error rate (QV)), and continuity (NG50/LG50 and NGA50/LGA50, Table 2, Additional file 1: Table S3). For the *A. thaliana* and *D. melanogaster* genomes, since the structure of these two genomes is relatively simple, most assemblers produced good assemblies. Notably, NextDenovo, Necat, and Flye outperformed Canu and Wtdbg2 on the overall evaluation metric, while NextDenovo, Necat, and Flye reported similar values for completeness and continuity. Concerning accuracy, compared to Necat and Flye, the NextDenovo assemblies contained fewer misassemblies and had a higher QV on the *D. melanogaster* genomes, although it exhibited two more misassemblies and a slightly smaller QV than the Flye assembly on the *A. thaliana* genome. In contrast, the genomes of *O. sativa* and *Z. mays* contain more repeats and are more complex, making them more challenging to assemble. Benefiting from the high accuracy of the error-corrected data, NextDenovo is able to distinguish different repeats more reliably, ensuring that the NextDenovo assemblies exhibit greater continuity than other assembler results, especially for the *Z. mays* genome. NextDenovo can deliver an assembly with about 2, 61, 15, and 758 times the NGA50 values of Necat, Canu, Flye, and Wtdbg2, respectively. Moreover, the NextDenovo assemblies also contained the smallest number of misassemblies and had a higher QV than the other tools. Regarding completeness, the assemblies produced by NextDenovo, Necat, Canu and Flye exhibit similar values in terms of assembly size and gene completeness. In fact, for the genomes of *A. thaliana* and the *O. sativa*, NextDenovo provided near-chromosome level assemblies, and since the LGA90 values for these two assemblies were only 10 and 20, it implied that most of the chromosomes contain only 1–2 long contigs.

**Table 2:** Statistics of nonhuman assemblies

Sample	Software	Assembly size (Mb)	NG50 (Mb)/LG50	NGA50 (Mb)/LGA50	No. of misassemblies	QV	Gene completeness (%)	Wall clock time (hour)
*A. thaliana* (452X)	NextDenovo	128.37	**15.18/5**	**15.18/5**	19	33.25	**99.20**	6.83
	Necat	124.55	15.01/5	14.98/5	44	31.93	**99.20**	6.82
	Canu	138.29	9.31/5	9.31/6	430	25.09	**99.20**	312.13
	Flye	121.16	14.63/5	14.63/5	**17**	**35.65**	**99.20**	12.00
	Wtdbg2	157.75	2.68/14	1.87/19	326	19.78	94.80	**2.10**
*D. melanogaster* (62X)	NextDenovo	134.34	18.11/4	15.68/4	**196**	**30.99**	98.70	1.07
	Necat	144.01	**19.55/4**	15.90/4	1,200	25.86	98.70	2.45
	Canu	154.94	8.58/6	5.68/7	1,738	23.53	**98.80**	45.55
	Flye	135.82	18.89/4	**17.32/4**	335	29.97	**98.80**	1.58
	Wtdbg2	137.49	6.32/7	5.33/9	919	26.07	97.20	**0.57**
*O. sativa* (230X)	NextDenovo	392.56	**30.55/6**	**18.00/9**	**81**	**26.45**	98.60	13.05
	Necat	394.40	25.44/7	17.86/9	183	25.83	**98.70**	10.85
	Canu	395.23	11.57/13	9.41/15	204	24.94	**98.70**	728.78
	Flye	403.45	11.10/14	7.84/18	115	24.76	**98.70**	25.02
	Wtdbg2	488.33	0.96/88	0.81/95	553	17.90	94.10	**5.85**
*Z. mays* (51X)	NextDenovo	2,118.82	**44.44/17**	**37.90/21**	**700**	**20.74**	**98.20**	**75.90**
	Necat	2,171.54	22.76/32	17.71/38	3,307	20.41	**98.20**	87.87
	Canu	2,240.87	0.65/950	0.62/995	6,284	19.14	98.10	1,741.77
	Flye	2,122.73	2.87/222	2.59/242	863	20.63	**98.20**	-
	Wtdbg2	4,068.86	0.07/11298	0.05/13848	22,258	14.07	97.00	-

NG50 is the length *N* that 50% of the reference genome is covered in contigs with length ≥ *N*. LG50 is the number of contigs with length ≥ NG50. NGA50 is an NG50 of aligned blocks that are obtained by breaking contigs at misassembly events and removing all unaligned bases. LGA50 is the number of aligned blocks with length ≥ NGA50. Misassemblies and QV are evaluated by QUAST, where QV is defined as $-10\times {{\text{log}}}_{10}(\frac{\# {\text{mismatches}} {\text{per}} 100 {\text{kbp}} + \# {\text{indels}} {\text{per}} 100 {\text{kbp}}}{100 {\text{kbp}}})$. Gene completeness is represented by the complete BUSCO values. QV and gene completeness were evaluated using the polished assemblies and other metrics were evaluated using the raw assemblies. The genomes of *A. thaliana*, *D. melanogaster*, and *O. sativa* were assembled on the same computer with 60 CPUs and 504 GB RAM of memory. The *Z. mays* genome, assembled by NextDenovo, Necat, and Canu, was run on a computer cluster with 7 nodes each with 32 CPUs and 256 GB RAM and assembled by Fly and Wtdbg2 run on a fat computer node. Best results for each metric are highlighted in bold

In terms of running time, NextDenovo is faster than Canu and Flye for the small (*D. melanogaster* and *A. thaliana*) or medium-sized genomes (*O. sativa*). For the repeat-rich *Z. mays* genome, NextDenovo was 23 times faster than Canu and slightly faster than Necat, but slower than Flye due to the limitations of the CTA algorithm. Notably, Wtdbg2 was the fastest among all the tools. It should be noted that the time consumption may vary if different parameters are used. In addition, NextDenovo can distribute almost all subtasks to run in parallel on computer cluster, and a subtask typically only required only 32 ~ 64 GB of peak memory. For most genomes, NextDenovo can complete genome assembly in a day when running on dozens of computer nodes.

To test the performance of different modules of NextDenovo, we used hybrid strategies that combined either the error correction step of NextDenovo with the assembly steps using the ATC-based tools Wtdbg2 and Flye (both tools can accept error-corrected reads as input) or the error correction step of Canu and Necat with the assembly step using NextDenovo. Overall, with a few exceptions, when combined with reads corrected by any tools as input, Wtdbg2 and Flye generally produced more contiguous assemblies than using raw data, and for relatively less complex genomes (*A. thaliana* and *D. melanogaster*), NextDenovo and Flye reported similar assemblies, better than Wtdbg2, and for complex genomes (*O. sativa* and *Z. mays*), NextDenovo reported much better assemblies than Wtdbg2 and Flye. In addition, Wtdbg2 assemblies using error-corrected reads from NextDenovo were generally more contiguous than those using error-corrected reads from Canu and Necat, with the exception of the *O. sativa* genome. The Flye assemblies using the error-corrected reads from NextDenovo were more contiguous on the relatively less complex genomes than those using the error-corrected reads from Canu and Necat but were more fragmented on the complex genomes. When replacing the assembly steps of Canu or Necat with NextDenovo, NextDenovo reported better assemblies than Canu on all test genomes, and NextDenovo produced similar assemblies to Necat on the *A. thaliana* and *O. sativa* genomes, better assembly than Necat on the *Z. mays* genome, but worse assembly than Necat on the *D. melanogaster* genome (Additional file 1: Table S4 and S5).

### Assembly of 35 human genomes by NextDenovo and comparative analysis of segmental duplications between humans

**Fig. 2:**
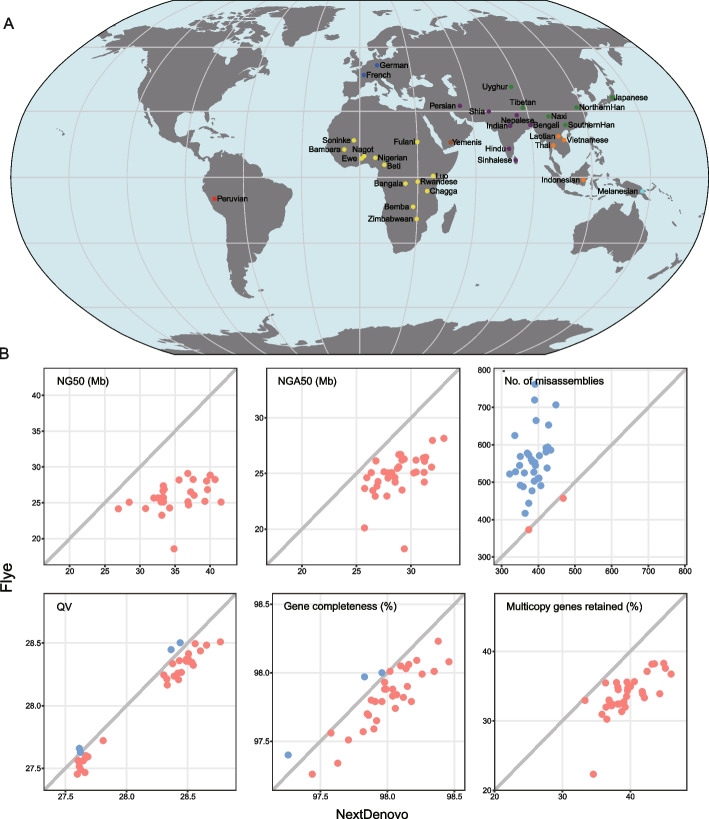
De novo assembly of 35 human genomes. **A** Geographical location of the 35 individuals sequenced. **B** Comparison of 35 human assemblies between NextDenovo and Flye. NG50 is the length *N* such that 50% of the reference genome is covered in contigs with length ≥ *N*. LG50 is the number of contigs with length ≥ NG50. NGA50 is NG50 of the aligned blocks that are obtained by breaking contigs at misassembly events and removing all unaligned bases. LGA50 is the number of aligned blocks with length ≥ NGA50. Misassemblies and QV were evaluated by QUAST, where QV is defined as $-10\times {{\text{log}}}_{10}(\frac{\# {\text{mismatches}} {\text{per}} 100 {\text{kbp}} + \# {\text{indels}} {\text{per}} 100 {\text{kbp}}}{100 {\text{kbp}}})$. Gene completeness and “multicopy genes retained” are reported by asmgene; “multicopy genes retained” corresponds to the percentage of multicopy genes in the reference genome that remains multicopy genes in the assembly. QV, gene completeness, and “multicopy genes retained” were evaluated using the polished assemblies and other metrics were evaluated using the raw assemblies. The metrics represented by the red points are larger than the metrics represented by the blue points

We envisage that the NextDenovo program will potentiate population-scale long-read assemblies, which in turn will facilitate the construction of human pan-genome using Nanopore long-read sequencing at low cost. Here, we collected blood samples from 35 humans with diverse ethnicities, including 13 from Africa, six from East Asia, four from Southeast Asia, six from South Asia, two from the Middle East, two from Europe, one from Oceania, and one from America (Fig. 2A, Additional file 1: Table S6 and S7). Principal component analysis (PCA) based on single nucleotide polymorphisms (SNPs) with the integration of the 1000 Genomes Project dataset indicated that the 35 genomes together covered much of the genetic diversity present in modern humans (Additional file 1: Fig. S2). For each individual, > 150 Gb long reads (mean length 21 kb) were sequenced using the Oxford Nanopore long-read sequencing platform. Each individual contained approximately 12,615 (~ 0.49 × in coverage) ultra-long reads (> 100 kb), which enabled a contiguous assembly of complex regions in the human genome [7, 8, 10, 23]. In addition, for each individual, ~ 150 Gb of short reads (100 bp) were sequenced for error polishing and correction.

Given Flye performed well in both simulated and real non-human data, we evaluated the performance of NextDenovo and Flye, representing two assembly strategies (CTA and ATC), for human genome assembly (Fig. 2B, Additional file 1: Table S7). On average, NextDenovo and Flye produced similar assembly sizes (2.83 Gb) with about 90.84% genome coverage, but the assemblies produced by NextDenovo covered more single-copy genes (97.99% vs. 97.82%) and retained more multi-copy genes (39.60% vs. 33.93%) than the Flye assemblies (Additional file 1: Table S7 and S8). Moreover, as with the results of the maize and rice genome assemblies, the NextDenovo assemblies contained longer (1.03–1.61-fold larger NGA50) and fewer contigs (68.18–96.97% of LGA50) than the Flye assemblies for all 35 genomes. More importantly, the NextDenovo assemblies contained 388 misassemblies on average, ~ 70% of that of the Flye assemblies, while the NextDenovo assemblies also had a slightly larger average QV than the Flye assemblies (28.17 vs. 28.06).

**Fig. 3:**
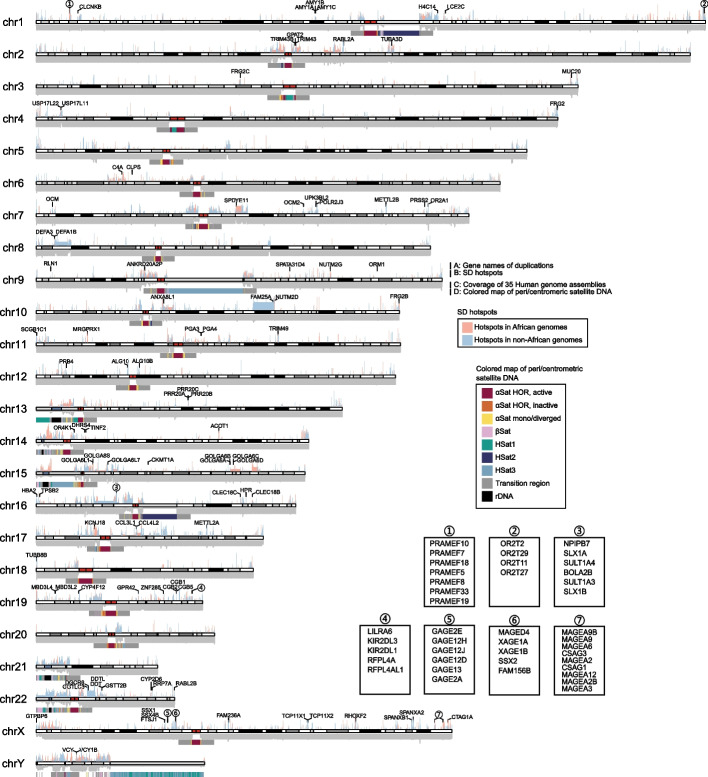
Distribution of duplicate genes and SD hotspots. **A** Gene symbols within duplications (gene names are marked by numbers and are shown in the subfigures). **B** Bar plots of SD hotspots in African/non-African genomes. **C** Coverage plot of 35 human genome assemblies. **D** Colored map of peri/centromeric satellite DNA (αSat: alpha satellite DNA, βSat: beta satellite DNA, HSat: human satellite DNA; see [10] for more detailed definitions). Ideogram plot was built from the T2T-CHM13 (v2) genome. Annotations of peri/centromeric and cytoband regions were downloaded from UCSC (https://hgdownload.soe.ucsc.edu/gbdb/hs1/)

Segmental duplications (SDs) are complex segments of DNA with near-identical sequences that are difficult to assemble by short reads; they nevertheless constitute important sources of structural diversity in the human genome and are associated with various human diseases [24, 25]. The use of long-read genome assembly techniques has facilitated the detection of SDs [25, 26]. Here, by using the “Brisk Inference of Segmental duplication Evolutionary structure” (BISER) [27], we identified an average of 133.6 Mbp of non-redundant SD sequences per individual (Additional file 1: Table S9), corresponding to ~ 4.7% of the human genome. Our results showed a notable correlation between total SD size and genome size (*R*^2^ = 0.9641, *p* < 2.2e − 16, Additional file 1: Fig. S3). We further identified African-specific SD hotspots, based on the difference of SD frequency between African and non-African assemblies (see the “ Methods” section). Our results showed that the highly differentiated hotspots were enriched in the pericentromeric regions (Fig. 3), which concurs with the predicted hotspots of genomic instability noted in T2T-CHM13 [25].

Long-read assembly holds out the promise of the comprehensive discovery of segmental duplications, especially the duplicated genes involved in SDs [25, 26]. We reasoned that these high-quality assemblies should facilitate the detection of gene duplications (Fig. 3 and Additional file 2: Table S10). In particular, we identified gains of salivary amylase (*AMY1*) gene copies with open reading frames and multiple exons in ten individuals (including 8 Asians and 2 Africans). For example, two individuals sampled from Vietnam and Thailand acquired four and three additional *AMY1* genes, respectively, which may have served to improve their ability to digest starchy foods such as rice. Indeed, the acquisition of additional copies of the *AMY1* gene is known to be a characteristic of populations with a high-starch diet [28], especially East and South East Asians. Additionally, four clusters of gene families, including preferentially expressed antigen of melanoma (PRAME), olfactory receptor (OR), G antigen (GAGE), and melanoma-associated antigen (MAGEA), exhibited dense clusters of SDs with paralogous genes (Fig. 3). Therefore, long-read sequencing makes it possible to accurately assemble those genomic regions that are characterized by highly similar paralogous clusters, including those containing expanded tandemly duplicated genes. Unfortunately, because we ran out of blood samples during the sequencing, additional experimental validation of the segmental duplications and duplicated genes was not possible.

## Discussion

NextDenovo is not only an accurate error-correction tool but also an efficient de novo assembler, specifically developed for noisy long reads using the CTA strategy. In our evaluation, NextDenovo was able to correct reads at a faster speed and generate more accurate corrected reads than Canu and Necat. The corrected reads usually have similar accuracy to the HiFi reads while maintaining the contiguity of the raw reads. For assembly, NextDenovo is much faster than the widely used CTA assembler, Canu. It is at least as fast or faster than Necat based on different input data. For the small and medium-sized genomes, it achieved a faster speed than Flye, but NextDenovo was usually slower than other ATC-based tools for large repeat-rich genomes due to the additional time-consuming error-correction step. However, on the other hand, with the high accuracy imparted by this error-correction step, NextDenovo can generate higher continuous assemblies containing fewer misassemblies. This is particularly true when assembling ONT “ultra-long” reads, since NextDenovo can generate partial or near chromosome-level assemblies, and this applies not only to human genome assembly but also to the assembly of complex plant genomes. Indeed, NextDenovo has been successfully applied to large genome assemblies several times, such as with the ~ 10.5 Gb *Cycas panzhihuaensis* genome (contigs N50 = 12 Mb) [29], the ~ 10.76 Gb allohexaploid oat genome (contig N50 = 75.27 Mb) [30], the ~ 40 Gb African lungfish genome (contig N50 = 1.60 Mb) [31], and the ~ 48 Gb Antarctic krill genome (contig N50 = 178.99 kb) [32]. Using ONT “ultra-long” reads, NextDenovo can generate partial or near chromosome-level assemblies. Thus, for the ~ 4.59 Gb papaver genome [33], NextDenovo produced an assembly with a contig N50 of 65.57 Mb, the longest length being 178.776 Mb using ~ 19X ONT “ultra-long” reads and ~ 86X ONT regular reads. In a similar vein, for the 3.69 Gb watermelon genome [34], NextDenovo produced an assembly in which the 11 longest contigs represent 11 chromosomes using ~ 57X ONT “ultra-long” reads. Finally, for the ~ 10.76 Gb allohexaploid oat genome [30], NextDenovo produced an assembly with a contig N50 of 75.27 Mb, the longest length being 313.87 Mb using ~ 100X ONT “ultra-long” reads.

Currently, we noticed that NextDenovo can be used for HiFi data assembly, but its assembly quality is significantly lower than Hifiasm (Additional file 1: Table S1 and Table S11) [4], an assembly tool developed specifically for HiFi data. Additionally, NextDenovo cannot be used for haplotype-resolved de novo genome assembly without trio binning due to sequencing errors, although it can detect the LSRs caused by heterozygosity, which is an advantage of assembly with HiFi data. However, ONT is gradually updating with new base calling models and chemistries that can improve the accuracy of raw reads, which should eventually make it possible for NextDenovo to perform haplotype-resolved assembly.

## Conclusions

NextDenovo is a highly efficient error correction and assembly tool for noisy long reads. It can quickly deliver highly accurate error-corrected reads and produce accurate assemblies from these reads. Especially when assembling with ONT “ultra-long” reads, NextDenovo can generate partial or near chromosome-level assemblies. Furthermore, NextDenovo is an excellent assembly tool for population-scale long-read assembly using Nanopore long-read sequencing data.

## Methods

We present the details of the algorithms of NextDenovo and the methods used in this study.

### Overview of the algorithms underlying the NextDenovo

NextDenovo consists of five main steps. The initial step involves pairwise raw read overlapping, followed by the second step which filters the overlapping results to avoid erroneous alignments that affect the error correction accuracy. The third step focuses on error correction based on the filtered overlapping results, while the fourth step entails a two-step iterative pairwise corrected reads overlapping. The final step involves constructing an assembly graph using the overlapping results, followed by graph cleaning and result outputting.

### Alignment and filtering

NextDenovo extracts the ~ 45X longest reads as seeds and performs pairwise reads overlapping all input reads and seeds using Minimap2 [35]. For each seed, NextDenovo partitions it into windows of 64 bp and calculates the overlapping depth in windows. A repeat window is defined as a window if its depth is greater than twice the average depth. A chimeric window is defined as a window if its depth is less than three. NextDenovo filters out an alignment if it is completely within a repeat window and splits a seed if it has a chimeric window.

### Error correction and LSR detection

NextDenovo first uses the KSC algorithm to perform the initial rough correction. The KSC algorithm is adapted from the Falconsense algorithm [36]; it calculates a confidence score using the following formula:

$${\text{score}}\left(P, b\right)={\text{max}}\left\{{\text{score}}\left(P-1, b\right)+{{\text{count}}}_{3-{\text{mer}}}\right\}-C$$

where *C* represents the valid depth at position *P*, $b\in \left\{A,T,G,C,-\right\}$, and then determines the correct path using a traceback procedure which starts at the last position *P*. Meanwhile, it records the low-quality positions where the chosen alleles account for ≤ 50% of the total. For each low-quality position, NextDenovo extends it on both sides until there are ≥ 16 consecutive non-low-quality positions. This extended region is defined as a low-score region (LSR) if it contains ≥ 4 low-quality positions.

### LSR correction

For an LSR *R* from a seed *S*, all subsequence *B*s that span this LSR from the overlapping reads of *S* are collected, and a kmer set (*K* = 8) at the 40 bp flanking sequences of *R* is produced. Then, for each *B*, the count of shared kmers between the kmer set from *B* and *R* is calculated as its matched kmer score. NextDenovo sorts all kmer scores of *B*s from large to small and removes all *B*s with a kmer score ≤ *C*, where *C* is half of its previous kmer score. For the KSC algorithm, deletion errors in the reference sequence are more harmful than insertion errors because the overlapping reads in the regions with insertion errors are not aligned. NextDenovo uses a greedy POA consensus algorithm that adopts a greedy strategy to insert bases in the consensus step to generate a pseudo-LSR seed by using the largest six *B*s ranked by kmer score. All pseudo-LSR seeds from *S* are linked to a long pseudo seed *L*, and all *B*s from *S* are mapped to *L*, and the KSC algorithm is applied to produce a corrected pseudo seed *P*. This procedure is called twice to improve the accuracy of the LSRs.

### Graph construction and cleaning

NextDenovo uses two rounds of pairwise overlapping to identify dovetail alignments using a modified Minimap2 between corrected seeds. The first round uses a large batch size and a large repetitive minimizer filtering threshold to rapidly detect candidate dovetail alignments. Then, for each candidate dovetail alignment, Minimap2 is used again with a smaller repetitive minimizer filtering threshold to produce more accurate alignments. Next, a directed string graph is constructed and transitive edges are removed. NextDenovo calculates the average indegree *I* and outdegree *O* of all nodes and clusters nodes into two categories, repeat nodes and non-repeat nodes. The repeat nodes are defined as nodes with indegree ≥ 1.5*I* or outdegree ≥ 1.5*O*, whereas other nodes are defined as non-repeat nodes. For the paths comprising only non-repeat nodes, the “best overlap graph” (BOG) algorithm is used to remove ambiguous edges. For repeat nodes, NextDenovo first calculates the maximum overlapping identity *I* and maximum overlapping length *L*, and maximum transitive score *S* (for an edge *E* from *a* to *c*, if there is node *b*, and there is an edge from *a* to *b* and an edge from *b* to *c*, then the count of *b* is defined as the transitive score of *E*) of out-edges or in-edges, and then removes any edges with overlapping identity <  = *i* x *I* and overlapping length *l* x *L* and transitive score *0.5* × *S* (here *i* and* l* are parameters). Subsequently, tips are removed and bubbles are resolved. Finally, for the complex subgraphs which usually contain many repeat nodes connected by only one in-node and one or more out-nodes, or one or more in-nodes and only one out-node, NextDenovo uses a series of gradually increasing overlapping identity, overlapping length, and transitive score thresholds to remove edges while maintaining connectivity between in-nodes and out-nodes.

### Evaluating error correction

To evaluate the performance of NextDenovo error correction, we simulated about 62X ONT data with an N50 length of 20.77 kb from chromosome 1 of the GRCh38 genome using NanoSim (v2.6.0) [37] and randomly extracted about 72X ONT data with N50 length of 56.77 kb from the chromosome 1 of the CHM13 genome (Additional file 1: Table S1). We next ran NextDenovo, Consent, Canu, and Necat with the same minimum read lengths to ensure consistency. Finally, we used minimap2 (-x map-ont) to map the corrected data to the reference and assessed their accuracy.

### Evaluating assemblies

We used QUAST for assembly evaluation. For the *A. thaliana*, *D. melanogaster*, and *Z. mays* datasets, we used appropriate NCBI assemblies as the reference genome. For the *O. sativa* dataset, we used the assembly of HiFi data from the same individual by hifiasm (v0.16.1) as the reference genome. For the human datasets, we used the T2T assembly of CHM13 as the reference genome. The assemblies were further polished with NextPolish using short and long reads and these genomes were subsequently used to evaluate QV and gene completeness. Gene completeness was evaluated with BUSCO for the *A. thaliana*, *D. melanogaster*, *O.sativa*, and *Z. mays* assemblies and paftools (v2.24) asmgene function [4] for the human assemblies. The commands and parameters used in this study are provided in the supplementary information file.

### Sample collection, DNA extraction, library preparation, and sequencing by Nanopore

Peripheral blood samples (~ 5 mL) were collected from people living in China. High-quality genomic DNA was extracted using the SDS (sodium dodecylbenzene sulfonate) method followed by purification with a QIAGEN® Genomic kit (Cat#13,343, QIAGEN) according to the standard procedures provided by the manufacturer. DNA degradation and contamination of the extracted DNA was monitored on 1% agarose gels. DNA purity was then detected using a NanoDrop™ One UV–Vis Spectrophotometer (Thermo Fisher Scientific, USA), with OD260/280 ranging from 1.8 to 2.0 and OD260/230 ranging from 2.0 to 2.2. Lastly, DNA concentration was measured using a Qubit® 3.0 Fluorometer (Invitrogen, USA).

In total, 2 μg DNA per sample was used as input material for the ONT (Oxford Nanopore Technologies) library preparations. After the DNA quality was controlled, size-selection of long DNA fragments was performed using the BluePippin system (Sage Science, USA). The DNA fragments were then end-repaired, and an A-ligation reaction was conducted using a NEBNext Ultra II End Repair/dA-tailing Kit (Cat# E7546). The adapter in an LSK109 kit was used for further ligation and the Qubit® 3.0 Fluorometer (Invitrogen, USA) was used to quantify the size of the library fragments. Sequencing was then performed on a Nanopore PromethION sequencer (Oxford Nanopore Technologies, UK) at Grandomics Biosciences Co. (Wuhan, China). The output FAST5 files of Nanopore sequencer containing signal data and base calling were converted to FAST5 files in FASTQ format with Guppy. The raw reads in fastq format with mean_qscore_template < 7 were then filtered out, resulting in pass reads.

### Library preparation and sequencing by MGISEQ2000

Genomic DNA (1 μg) was randomly fragmented by Covaris. The fragmented DNA was selected by an Agencourt AMPure XP-Medium Kit to an average size of 200–400 bp. The selected fragments were subjected to end repair, 3′ adenylation, adaptor ligation, and polymerase chain reaction (PCR) amplification, with the products, and then being recovered using an AxyPrep Mag PCR Clean-up Kit. The double-stranded PCR products were heat denatured and circularized by the splint oligo sequence. Single-stranded circular DNA (ssCir DNA) was formatted as the final library and quality controlled. The quality-controlled libraries were sequenced on the MGISEQ2000 platform.

### Diversity of 35 human genomes

To determine the diversity of 35 human genomes, we mapped the short reads to the GRCh38 reference assembly using the BWA-MEM (v0.7.15) algorithm [38]. After sorting the reads by coordinates, and removing duplicate reads using SAMtools (v1.8) [39], HaplotypeCaller and CombineGVCFs in the Genome Analysis Toolkit (GATK, v4.0.4.0) [40] were used for calling and combining the GVCF files. We then applied the GenotypeGVCFs method in GATK to genotype SNPs based on genome positions from the 1000 Genomes Project dataset [41, 42]. After SNP filtering with “QUAL < 50,” we merged the SNPs with the 1000 Genomes Project data for principal component analysis.

### Gene, gene duplications, and repeat annotations

Gene annotations of the 35 human genomes were performed by mapping GENCODE (v35) annotations [43] from GRCh38 using Liftoff (v1.6.3) [44] with the following settings: liftoff -flank 0.1 -sc 0.85 -copies. Duplicate genes were identified based on the following criteria: (1) extra copy number > 1, (2) the number of exons > 1, (3) CDS length > 200 bp, and (4) containing complete open reading frames (ORF). Repeat annotations were conducted with RepeatMasker (v4.1.3) [45] and Tandem Repeats Finder (TRF) [46]. RepeatMasker was run with default settings and TRF was run with “trf 2 7 7 80 10 50 15 -l 25 -h -ngs” parameters.

### Segmental duplication (SD) analysis

SDs were identified using BISER (v1.2.3) [27] based on the soft-masked human genomes. Low-quality SDs were filtered out using the following criteria: (1) < 1 kbp in length; (2) > 70% overlapping with satellite sequence or > 10% overlapping with simple repeats annotated with RepeatMasker; (3) < 90% identical by gap-compressed identity or < 50% identical including indels. The pipeline was conducted using an R script (open access on GitHub) [47] and a modified snakemake file downloaded from GitHub [48]. Next, we annotated 35 human genomes with unique ancestral units (duplicons) identified by DupMasker [49]. Regions that do not overlap with the duplicons were annotated as new SDs. Finally, we defined the African-specific SD hotspots based on the frequency difference of SDs between African and non-African assemblies. The specific calculation steps were as follows: (1) obtained the non-redundant SD regions for each human assembly, (2) calculated the frequency of SD coverage within African and non-African groups, (3) computed the difference between the frequency of African and non-African of SDs. Regions with a difference much greater than zero were defined as African-specific SD hotspots. We mapped the positional information of SDs from 35 human genome assemblies to the T2T-CHM13-v2.0 [50] genome using the “paftools liftover” tool for visualization. SDs hotspots calculation and visualization were carried out with R packages: tidyverse [51], rtracklayer [52], plyranges [53], and karyoploteR [54].

### Supplementary Information

**Additional file 1: Fig. S1**. The overlap of corrected reads between Canu, Consent and NextDenovo. **Fig. S2**. Principal component analysis of 35 individuals integrated with the human 1000 Genomes Project data. **Fig. S3**. Linear regression between total segmental duplication (SD) size and genome size. **Table S1**. Statistical information of the six ONT datasets used in this study. **Table S2**. Statistics of ONT simulation read error correction. **Table S3**. BUSCO scores of non-human assemblies. **Table S4**. Statistics of nonhuman assemblies using hybrid strategies. **Table S5**. BUSCO scores of non-human assemblies using hybrid strategies. **Table S6**. Sample information of 35 human samples. **Table S7**. Statistics of 35 human assemblies. **Table S8**. Gene completeness of 35 human assemblies. **Table S9**. Summary statistic of segmental duplications across 35 human assemblies. **Table S11**. Statistics of assemblies with HiFi data.

**Additional file 2: Table S10**. Duplicate gene annotation across 35 human assemblies.

**Additional file 3. **Software commands used in this study.

**Additional file 4. **Review history.

## Data Availability

The ONT dataset and reference genome for CHM13 were obtained from GitHub [55]. The ONT, short reads dataset, and reference genome for *A. thaliana* were downloaded from BIG Data Center, Beijing Institute of Genomics (BIG), Chinese Academy of Sciences, under accession no. PRJCA005809 (Bioproject), CRR302667 (ONT), CRR302670 (short reads), and GWHBDNP00000001.1 (reference genome) [56]. The datasets were obtained from the NCBI Sequence Read Archive: SRR6702603 and SRR6821890 as ONT dataset, SRR6702604 as short reads dataset for *D. melanogaster* [57], SRR10948639-SRR10948642 as ONT dataset, SRR10948643 as HiFi dataset, SRR10948638 as short reads dataset for *O. sativa* [58], SRR12482959-SRR12482969 as ONT dataset, SRR11870962 as short reads dataset for *Z. mays* [59]. The reference genomes of *D. melanogaster* and *Z. mays* were downloaded from the NCBI GenBank under accession no. GCA_000001215.4 [60] and GCA_014529475.1 [59], respectively. All human data, including raw data and de novo assemblies, were deposited at the Genome Sequence Archive for Human on the National Genomics Data Center, China National Center (NGDC) for Bioinformation/Beijing Institute of Genomics, Chinese Academy of Sciences, under BioProject ID of PRJCA006287 [61] (accession number: HRA004135). Sample collection and data release are permitted by the Ministry of Science and Technology of the People’s Republic of China (permission no. 2021BAT3787). The raw sequencing data of Chinese individuals are available but with restricted access. For more detailed guidance on accessing the data, please refer to the Implementation Rule for the Regulations of the People’s Republic of China on Administration of Human Genetic Resources [62] and the GSA-Human Data Access Request Guide for Users [63]. Any organization or individual out of China to receive and use Human Genetic Resources should collaborate with Chinese entities. We can assist in submitting the application and obtaining the approval from the Human Genetic Resources Administration of China. NextDenovo code are available on GitHub [64] or Zenodo [65] under GNU GPLv3 license, and benchmarking data are available on Read the Docs [66]. The codes and intermediate data of SD analysis are publicly available on Zenodo [67].
