# Supplementary material for: NextDenovo: an efficient error correction and accurate assembly tool for noisy long reads
Source: Genome Biol. 2024 Apr 26;25:107. doi: 10.1186/s13059-024-03252-4 (PMC11046930; doi:10.1186/s13059-024-03252-4)
Supplement: Supplementary file 1 — Additional file 1: Fig. S1. The overlap of corrected reads between Canu, Consent and NextDenovo. Fig. S2. Principal component analysis of 35 individuals integrated with the human 1000 Genomes Project data. Fig. S3. Linear regression between total segmental duplication (SD) size and genome size. Table S1. Statistical information of the six ONT datasets used in this study. Table S2. Statistics of ONT simulation read error correction. Table S3. BUSCO scores of non-human assemblies. Table S4. Statistics of nonhuman assemblies using hybrid strategies. Table S5. BUSCO scores of non-human assemblies using hybrid strategies. Table S6. Sample information of 35 human samples. Table S7. Statistics of 35 human assemblies. Table S8. Gene completeness of 35 human assemblies. Table S9. Summary statistic of segmental duplications across 35 human assemblies. Table S11. Statistics of assemblies with HiFi data. [file 13059_2024_3252_MOESM1_ESM.docx]

# Electronic Supplementary Material for “An efficient error correction and assembly tool for noisy long reads”

## Supplementary Figures


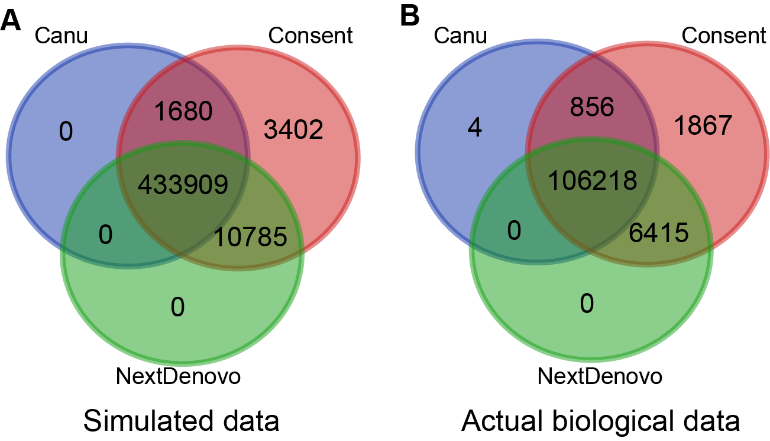


**Fig. S1. The overlap of corrected reads between Canu, Consent and NextDenovo.** The Necat correction reads have been renamed, so they cannot be used for this comparison.


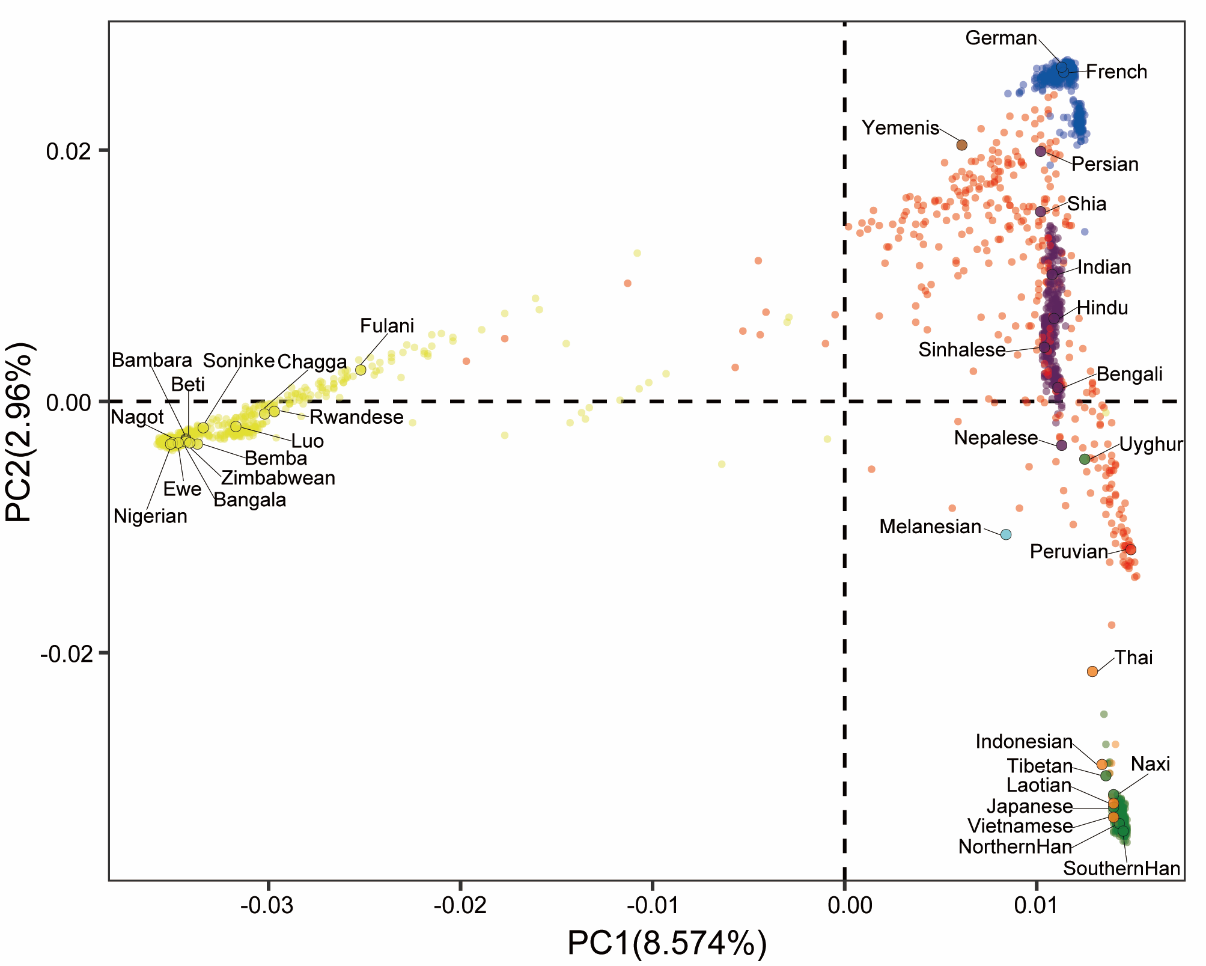


**Fig. S2. Principal component analysis of 35 individuals integrated with the human 1000 Genomes Project data.**


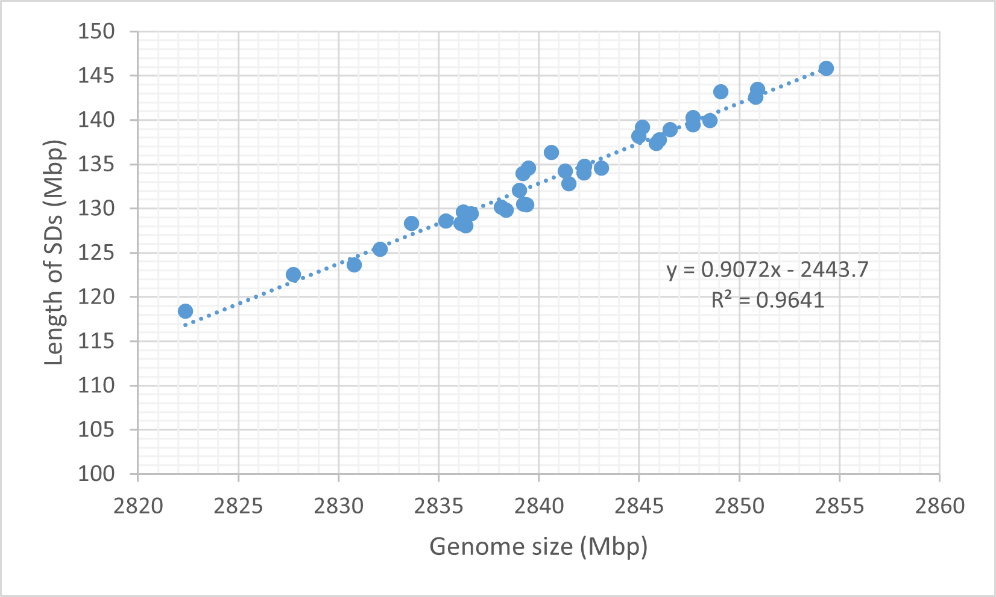


**Fig. S3. Linear regression between total segmental duplication (SD) size and genome size, with a significant correlation found between SD length and genome size (R^2^ = 0.9641, p < 2.2e-16).**

## Supplementary Tables

**Table S1: Statistical information of the six ONT datasets used in this study**

| **Datasets** | | **Bases (bp)** | **Read count** | **N50  (bp)** | **Average length (bp)** | **Base coverage** |
| --- | --- | --- | --- | --- | --- | --- |
| Simulation (chr1) | ONT | 15,356,858,115 | 889,516 | 20,769 | 17,264 | 61.68 |
| CHM13 (chr1) |  | 17,929,712,977 | 554,014 | 56,766 | 32,363 | 72.02 |
| *A. thaliana* |  | 56,814,196,989 | 3,064,191 | 46,452 | 18,541 | 454.51 |
| *D. melanogaster* |  | 8,965,907,711 | 1,145,050 | 11,980 | 7,830 | 62.26 |
| *O. sativa* |  | 92,929,730,682 | 6,100,295 | 41,473 | 15,233 | 232.32 |
| *Z. mays* |  | 118,058,481,710 | 8,278,142 | 19,840 | 14,261 | 51.33 |
| CHM13 | HiFi | 100,369,168,661 | 5,567,158 | 17,781 | 18,028 | 32.86 |
| *A. thaliana* |  | 22,904,700,074 | 1,517,433 | 15,424 | 15,094 | 183.24 |
| *D. melanogaster* |  | 5,479,585,807 | 443,920 | 12,360 | 12,343 | 38.05 |
| *O. sativa* |  | 19,964,422,872 | 1,494,013 | 13,586 | 13,362 | 49.91 |

**Table S2: Statistics of ONT simulation read error correction**

| **Source** | **Median read length (bp, parameter used by NanoSim)** | **Software** | **Corrected bases rate (%)** | **Average length (bp)** | **Max length (bp)** | **Reads with chimeric alignments  (%)** | **Mapped with ≥99% coverage (%)** | **Mapped with ≥97% identity (%)** | **Average error rate (%)** | **Wall clock time (hour)** | **Cpu time (hour)** | **Maximum RAM (GB)** |
| --- | --- | --- | --- | --- | --- | --- | --- | --- | --- | --- | --- | --- |
| NanoPore simulation data (chr1, 62X) | 10,000 | Raw Reads | - | 26,542 | 99,993 | **0.03** | 75.49 | 0.00 | 9.69 | - | - | - |
|  |  | NextDenovo | 83.61 | **26,911** | 100,015 | **0.03** | **99.96** | **99.96** | **0.05** | **1.67** | **47.62** | 17.83 |
|  |  | Necat | **84.27** | 26,909 | **100,565** | 0.09 | 99.84 | 99.76 | 0.25 | 3.02 | 58.31 | 37.57 |
|  |  | Canu | 80.97 | 27,267 | 99,778 | 0.27 | 99.40 | 98.91 | 0.70 | 20.17 | 442.45 | **12.19** |
|  | 20,000 | Raw Reads | - | 40,964 | 99,982 | 0.06 | 77.42 | 0.00 | 9.70 | - | - | - |
|  |  | NextDenovo | 84.58 | 41,654 | 100,010 | **0.05** | **99.95** | **99.97** | **0.05** | **1.75** | **51.16** | 17.81 |
|  |  | Necat | **85.06** | 41,725 | **100,885** | 0.11 | 99.82 | 99.71 | 0.28 | 3.07 | 60.67 | 37.68 |
|  |  | Canu | 81.89 | **41,927** | 99,713 | 0.43 | 99.30 | 99.04 | 0.66 | 28.93 | 576.17 | **12.17** |
|  | 30,000 | Raw Reads | - | 49,299 | 99,977 | 0.07 | 77.94 | 0.00 | 9.70 | - | - | - |
|  |  | NextDenovo | 84.72 | 50,228 | 100,013 | **0.06** | **99.94** | **99.97** | **0.05** | **1.82** | **53.03** | 17.90 |
|  |  | Necat | **85.15** | 50,342 | **100,709** | 0.10 | 99.82 | 99.71 | 0.29 | 3.08 | 61.80 | 37.74 |
|  |  | Canu | 82.20 | **50,345** | 100,120 | 0.45 | 99.30 | 99.15 | 0.63 | 36.17 | 654.08 | **12.16** |
|  | 50,000 | Raw Reads | - | 57,918 | 99,999 | 0.10 | 78.44 | 0.00 | 9.70 | - | - | - |
|  |  | NextDenovo | 84.85 | 59,013 | 100,081 | **0.07** | **99.93** | **99.98** | **0.04** | **1.83** | **53.45** | 17.87 |
|  |  | Necat | **85.26** | **59,161** | **101,182** | 0.13 | 99.78 | 99.70 | 0.29 | 3.22 | 63.87 | 37.77 |
|  |  | Canu | 82.47 | 58,950 | 100,074 | 0.56 | 99.21 | 99.11 | 0.62 | 40.87 | 755.65 | **12.15** |

Only the primary alignments defined by minimap2 of each read were used for evaluation. Corrected base rate is the ratio of the size of the corrected reads to the size of the raw reads to be corrected. Reads with chimeric alignments are defined as reads that have supplementary alignments. Average error rate only uses the reads that are mapped with ≥80% coverage. All the software was tested on the same computer with 32 CPUs and 252 GB RAM of memory. We failed to run Consent on these datasets due to a segmentation fault. Best results for each metric is highlighted in bold.

**Table S3: BUSCO scores of non-human** **assemblies**

| **Datasets (post-polish)** | **Assembler** | **Complete BUSCOs** | | **Fragmented BUSCOs (%)** | **Missing BUSCOs (%)** |
| --- | --- | --- | --- | --- | --- |
|  |  | **Single-copy (%)** | **Duplicated (%)** |  |  |
| *A. thaliana* | NextDenovo | 97.90 | 1.30 | 0.00 | 0.80 |
|  | Necat | 97.90 | 1.30 | 0.00 | 0.80 |
|  | Canu | 97.30 | 1.90 | 0.00 | 0.80 |
|  | Flye | 97.90 | 1.30 | 0.00 | 0.80 |
|  | Wtdbg2 | 92.10 | 2.70 | 0.20 | 5.00 |
| *D. melanogaster* | NextDenovo | 98.50 | 0.20 | 0.50 | 0.80 |
|  | Necat | 98.50 | 0.20 | 0.50 | 0.80 |
|  | Canu | 98.50 | 0.30 | 0.50 | 0.70 |
|  | Flye | 98.60 | 0.20 | 0.50 | 0.70 |
|  | Wtdbg2 | 97.00 | 0.20 | 0.50 | 2.30 |
| *O. sativa* | NextDenovo | 96.30 | 2.30 | 0.20 | 1.20 |
|  | Necat | 96.30 | 2.40 | 0.20 | 1.10 |
|  | Canu | 96.40 | 2.30 | 0.20 | 1.10 |
|  | Flye | 96.50 | 2.20 | 0.20 | 1.10 |
|  | Wtdbg2 | 90.10 | 4.00 | 0.60 | 5.30 |
| *Z. mays* | NextDenovo | 82.80 | 15.40 | 0.20 | 1.60 |
|  | Necat | 82.70 | 15.50 | 0.20 | 1.60 |
|  | Canu | 81.50 | 16.60 | 0.30 | 1.60 |
|  | Flye | 82.70 | 15.50 | 0.20 | 1.60 |
|  | Wtdbg2 | 67.30 | 29.70 | 0.70 | 2.30 |

**Table S4: Statistics of nonhuman assemblies using hybrid strategies**

| **Sample** | **Software (error correction)** | **Software (assembly)** | **Assembly size (Mb)** | **NG50 (Mb)/LG50** | **NGA50 (Mb)/LGA50** | **No. of misassemblies** | **QV** | **Gene completeness (%)** |
| --- | --- | --- | --- | --- | --- | --- | --- | --- |
| *A. thaliana* (455X) | **-** | Flye | 121.16 | 14.63/5 | 14.63/5 | 17 | 35.65 | 99.20 |
|  | NextDenovo |  | 132.25 | **16.49/4** | **16.49/4** | 12 | **38.11** | 99.20 |
|  | Necat |  | 121.64 | 14.97/4 | 14.78/5 | 31 | 32.77 | 99.20 |
|  | Canu |  | 118.81 | 14.40/5 | 14.17/5 | **5** | 37.97 | 99.20 |
|  | - | NextDenovo | 128.37 | **15.18/5** | **15.18/5** | **19** | **33.25** | 99.20 |
|  | Necat |  | 122.48 | 14.78/5 | 14.77/5 | 24 | 31.86 | 99.20 |
|  | Canu |  | 122.46 | 14.67/54 | 14.66/5 | 50 | 32.61 | 99.20 |
|  | - | Wtdbg2 | 157.75 | 2.68/14 | 1.87/19 | 326 | 19.78 | 94.80 |
|  | NextDenovo |  | 130.63 | **13.68/5** | **13.67/5** | 80 | 29.04 | 98.40 |
|  | Necat |  | 122.78 | 13.57/5 | 11.23/5 | **39** | **30.44** | 97.90 |
|  | Canu |  | 122.56 | 9.28/6 | 9.28/6 | 66 | 28.37 | **98.70** |
|  | - | Flye | 135.82 | **18.89/4** | **17.32/4** | 335 | 29.97 | **98.80** |
| *D. melanogaster* (62X) | NextDenovo |  | 141.70 | 17.71/4 | 15.65/4 | 1,289 | 26.32 | **98.80** |
|  | Necat |  | 137.68 | 17.68/4 | 15.64/4 | 669 | 28.52 | 98.70 |
|  | Canu |  | 129.08 | 1.55/21 | 1.52/22 | **98** | **33.62** | 98.50 |
|  | - | NextDenovo | 134.34 | **18.11/4** | **15.68/4** | 196 | 30.99 | **98.70** |
|  | Necat |  | 133.55 | 8.52/5 | 6.91/6 | 165 | **31.96** | **98.70** |
|  | Canu |  | 133.09 | 12.93/4 | 12.91/4 | **142** | 31.80 | 98.60 |
|  | - | Wtdbg2 | 137.49 | 6.32/7 | 5.33/9 | 919 | 26.07 | 97.20 |
|  | NextDenovo |  | 139.85 | **17.46/4** | **13.84/4** | 917 | 26.91 | **98.50** |
|  | Necat |  | 130.44 | 2.55/15 | 2.55/15 | 767 | **27.63** | 93.10 |
|  | Canu |  | 132.74 | 1.73/22 | 1.69/22 | **732** | 26.18 | 96.30 |
| *O. sativa* (232X) | - | Flye | 403.45 | 11.10/14 | 7.84/18 | 115 | 24.76 | **98.70** |
|  | NextDenovo |  | 378.13 | 9.84/16 | 8.73/17 | 47 | **28.16** | 98.60 |
|  | Necat |  | 381.77 | **11.55/14** | **10.42/15** | **31** | 26.22 | 98.60 |
|  | - | NextDenovo | 392.56 | **30.55/6** | **18.00/9** | 81 | 26.45 | 98.60 |
|  | Necat |  | 391.29 | 25.47/7 | 17.72/9 | **43** | 25.68 | **98.70** |
|  | Canu |  | 389.11 | 30.34/6 | 17.68/9 | 78 | **26.47** | 98.60 |
|  | - | Wtdbg2 | 488.33 | 0.96/88 | 0.81/95 | 553 | 17.90 | 94.10 |
|  | NextDenovo |  | 388.41 | 11.23/14 | 8.07/16 | 132 | 24.33 | 96.80 |
|  | Necat |  | 376.32 | **12.37/10** | **10.15/14** | **57** | **24.52** | **97.40** |
|  | Canu |  | 387.25 | 4.18/27 | 3.59/30 | 85 | 23.96 | 97.20 |
| *Z. mays* (51X) | - | Flye | 2,122.73 | 2.87/222 | 2.59/242 | 863 | 20.63 | 98.20 |
|  | NextDenovo |  | 2,116.55 | 9.06/70 | 8.31/75 | **474** | **20.79** | 98.20 |
|  | Necat |  | 2,116.72 | **20.66/37** | **16.75/40** | 583 | 20.77 | 98.20 |
|  | - | NextDenovo | 2,118.82 | **44.44/17** | **37.90/21** | 700 | **20.74** | **98.20** |
|  | Necat |  | 1,965.81 | 42.33/19 | 30.05/24 | **632** | 19.62 | 76.70 |
|  | Canu |  | 2,105.47 | 2.80/226 | 2.23/288 | 1113 | 18.84 | 97.8 |
|  | - | Wtdbg2 | 4,068.86 | 0.07/11298 | 0.05/13848 | 22,258 | 14.07 | **97.00** |
|  | NextDenovo |  | 2,194.10 | **0.50/1185** | **0.47/1252** | 2,032 | 19.58 | 94.40 |
|  | Necat |  | 2,001.89 | 0.44/1300 | 0.42/1336 | **1,280** | **20.02** | 92.00 |
|  | Canu |  | 2,180.39 | 0.19/3230 | 0.17/3525 | 4,013 | 16.61 | 96.50 |

NG50 is the length N such that 50% of the reference genome is covered in contigs with length ≥ N. LG50 is the number of contigs with length ≥ NG50. NGA50 is an NG50 of aligned blocks that are obtained by breaking contigs at misassembly events and removing all unaligned bases. LGA50 is the number of aligned blocks with length ≥ NGA50. Misassemblies and QV are evaluated by QUAST, where QV is defined as $-10\times\log_{10} (\frac{\# mismatches per 100 kbp + \# indels per 100 kbp}{100 kbp})$. Gene completeness is represented by the complete BUSCO values. QV and gene completeness were evaluated using the polished assemblies and other metrics were evaluated using the raw assemblies. Flye reports errors when assembling Canu-corrected reads from *O. sativa* and *Z. mays* datasets. Best results for each metric are highlighted in bold.

**Table S5: BUSCO scores of non-human assemblies using hybrid strategies**

| **Datasets (post-polish)** | **Software (error correction)** | **Software (assembly)** | **Complete BUSCOs** | | **Fragmented BUSCOs (%)** | **Missing BUSCOs (%)** |
| --- | --- | --- | --- | --- | --- | --- |
|  |  |  | **Single-copy (%)** | **Duplicated (%)** |  |  |
| *A. thaliana* (455X) | NextDenovo | Flye | 97.90 | 1.30 | 0.00 | 0.80 |
|  | Necat |  | 97.90 | 1.30 | 0.00 | 0.80 |
|  | Canu |  | 97.80 | 1.40 | 0.00 | 0.80 |
|  | Necat | NextDenovo | 97.90 | 1.30 | 0.00 | 0.80 |
|  | Canu |  | 97.90 | 1.30 | 0.00 | 0.80 |
|  | NextDenovo | Wtdbg2 | 94.30 | 4.10 | 0.10 | 1.50 |
|  | Necat |  | 94.60 | 3.30 | 0.10 | 2.00 |
|  | Canu |  | 97.10 | 1.60 | 0.10 | 1.20 |
| *D. melanogaster* (62X) | NextDenovo | Flye | 98.50 | 0.30 | 0.50 | 0.70 |
|  | Necat |  | 98.50 | 0.20 | 0.50 | 0.80 |
|  | Canu |  | 98.20 | 0.30 | 0.50 | 1.00 |
|  | Necat | NextDenovo | 98.40 | 0.30 | 0.50 | 0.80 |
|  | Canu |  | 98.40 | 0.20 | 0.50 | 0.90 |
|  | NextDenovo | Wtdbg2 | 98.30 | 0.20 | 0.50 | 1.00 |
|  | Necat |  | 92.80 | 0.30 | 0.50 | 6.40 |
|  | Canu |  | 96.00 | 0.30 | 0.50 | 3.20 |
| *O. sativa* (232X) | NextDenovo | Flye | 96.30 | 2.30 | 0.20 | 1.20 |
|  | Necat |  | 96.30 | 2.30 | 0.20 | 1.20 |
|  | Necat | NextDenovo | 96.50 | 2.20 | 0.20 | 1.10 |
|  | Canu |  | 96.40 | 2.20 | 0.20 | 1.20 |
|  | NextDenovo | Wtdbg2 | 94.50 | 2.30 | 0.20 | 3.00 |
|  | Necat |  | 95.10 | 2.30 | 0.20 | 2.40 |
|  | Canu |  | 95.00 | 2.20 | 0.20 | 2.60 |
| *Z. mays* (51X) | NextDenovo | Flye | 82.70 | 15.50 | 0.30 | 1.50 |
|  | Necat |  | 82.80 | 15.40 | 0.20 | 1.60 |
|  | Necat | NextDenovo | 52.40 | 24.30 | 0.90 | 22.40 |
|  | Canu |  | 82.40 | 15.40 | 0.30 | 1.90 |
|  | NextDenovo | Wtdbg2 | 76.70 | 17.70 | 0.60 | 5.00 |
|  | Necat |  | 77.30 | 14.70 | 0.70 | 7.30 |
|  | Canu |  | 79.50 | 17.00 | 0.50 | 3.00 |

**Table S6: Sample information of 35 human samples**

| No. | Sample | Country | Population | Super-population | Ethnic Group | Language | Gender | Latitude | Longitude | Depth | Depth (ChrX) | Coverage | Coverage (ChrX) |
| --- | --- | --- | --- | --- | --- | --- | --- | --- | --- | --- | --- | --- | --- |
| 1 | A01 | Benin | AFR | AFR | Nagot/Yaruba | Nagot | M | 9.62 | 2.34 | 46.47 | 24.78 | 0.93 | 0.96 |
| 2 | A03 | Mali | AFR | AFR | Bambara | Bambara | M | 17.36 | -3.53 | 45.70 | 24.29 | 0.93 | 0.95 |
| 3 | A04 | Tanzania | AFR | AFR | Chagga | Swahili | M | -6.31 | 34.85 | 46.01 | 24.56 | 0.93 | 0.96 |
| 4 | A05 | Cameroon | AFR | AFR | Beti | Eton | M | 5.69 | 12.72 | 42.83 | 22.77 | 0.93 | 0.96 |
| 5 | A06 | Congo | AFR | AFR | Bangala | Lingala | M | -2.88 | 23.66 | 41.93 | 22.56 | 0.93 | 0.96 |
| 6 | A07 | Mali | AFR | AFR | Soninke | Soninke | M | 17.36 | -3.53 | 42.31 | 22.56 | 0.93 | 0.96 |
| 7 | A08 | Rwanda | AFR | AFR | Rwandese | Kinyarwanda | M | -2.02 | 29.90 | 43.55 | 23.22 | 0.93 | 0.96 |
| 8 | A09 | Togo | AFR | AFR | Ewe | Ewe | M | 8.51 | 0.98 | 44.14 | 23.64 | 0.93 | 0.95 |
| 9 | A10 | N.Sudan | AFR | AFR | Fulani | Arabic | M | 16.09 | 30.09 | 45.16 | 24.14 | 0.93 | 0.96 |
| 10 | A11 | Zambia | AFR | AFR | Bemba | Bemba | M | -13.46 | 27.79 | 44.49 | 23.70 | 0.93 | 0.96 |
| 11 | A15 | Zimbabwe | AFR | AFR | - | - | F | -19.00 | 29.87 | 52.70 | 54.04 | 0.92 | 0.96 |
| 12 | Nigeria | Nigeria | AFR | AFR | - | - | M | 8.87 | 8.04 | 53.36 | 28.60 | 0.93 | 0.96 |
| 13 | Kenya | Kenya | AFR | AFR | - | - | M | 0.58 | 37.84 | 46.68 | 25.01 | 0.93 | 0.96 |
| 14 | Han | China | EA | EA | Han(North) | Chinese | M | 31.81 | 117.22 | 52.97 | 28.59 | 0.93 | 0.96 |
| 15 | LX | China | EA | EA | Han(South) | Chinese | M | 23.79 | 108.77 | 46.78 | 25.13 | 0.93 | 0.96 |
| 16 | Xinjiang | China | EA | EA | Uygur | - | M | 41.21 | 85.33 | 44.49 | 23.83 | 0.93 | 0.96 |
| 17 | Zang | China | EA | EA | Tibetan | - | M | 31.68 | 88.17 | 43.98 | 23.35 | 0.93 | 0.96 |
| 18 | Mo | China | EA | EA | Mosuo | - | M | 27.72 | 100.79 | 40.91 | 22.32 | 0.93 | 0.96 |
| 19 | Y10 | Japan | EA | EA | Buddist | Japanese | M | 36.28 | 139.08 | 38.57 | 20.74 | 0.93 | 0.96 |
| 20 | Iran | Iran | SA | SA | Persian | Persian/Farsi | M | 32.50 | 54.29 | 45.80 | 24.70 | 0.93 | 0.96 |
| 21 | Yin | India | SA | SA | - | - | M | 23.41 | 79.46 | 43.79 | 23.68 | 0.93 | 0.96 |
| 22 | Y9 | India/NE | SA | SA | Hindu | Hindi | M | 23.41 | 79.46 | 37.13 | 20.09 | 0.93 | 0.96 |
| 23 | Y5 | Nepal | SA | SA | Hind | Nepali | M | 28.26 | 83.94 | 52.77 | 27.87 | 0.93 | 0.96 |
| 24 | A12 | Bangladesh | SA | SA | Bengali | Bangla | M | 23.73 | 90.31 | 45.06 | 24.24 | 0.93 | 0.96 |
| 25 | A02 | Siri Lanka | SA | SA | Sinhala | Sinhala | F | 7.79 | 80.68 | 51.05 | 52.47 | 0.92 | 0.96 |
| 26 | Y3 | Pakistan | SA | SA | Muslim/shia | Sindhi/urdu | M | 29.92 | 69.36 | 48.71 | 25.92 | 0.93 | 0.96 |
| 27 | Y4 | Laos | SEA | SEA | Buddist | Lao | M | 18.65 | 104.15 | 44.74 | 23.87 | 0.93 | 0.96 |
| 28 | Y1 | Indonesia | SEA | SEA | Javanese | Bahasa Indonesia | M | -1.25 | 115.42 | 45.43 | 24.25 | 0.93 | 0.96 |
| 29 | Y6 | Vietnam | SEA | SEA | LE DUC HUAN | Vietnam | M | 16.94 | 106.82 | 40.96 | 21.65 | 0.93 | 0.96 |
| 30 | Y7 | Thailand | SEA | SEA | Buddist | THAI | M | 14.48 | 100.85 | 44.77 | 23.66 | 0.93 | 0.96 |
| 31 | E1 | France | EUR | EUR | - | French | M | 46.64 | 2.34 | 45.62 | 24.62 | 0.93 | 0.96 |
| 32 | Y8 | Germany | EUR | EUR | Caucasiau | German | F | 51.20 | 10.38 | 44.47 | 46.04 | 0.92 | 0.96 |
| 33 | Y2 | Yemen | WA | WA | Muslim | Arabic | M | 15.89 | 47.49 | 47.64 | 25.50 | 0.93 | 0.96 |
| 34 | Ba | Papua New Guinea | SWP | SWP | - | - | M | -6.40 | 146.11 | 43.67 | 23.06 | 0.93 | 0.96 |
| 35 | SA1 | Peru | AMR | AMR | - | - | M | -9.80 | -75.22 | 44.80 | 23.94 | 0.93 | 0.96 |

**Table S7: Statistics of 35 human assemblies**

| **Sample** | **Software** | **Assembly size (Gb)** | **NG50 (Mb)/LG50** | **NGA50 (Mb)/LGA50** | **No. misassemblies** | **QV** | **Gene completeness (%)** | **Multicopy genes retained (%)** |
| --- | --- | --- | --- | --- | --- | --- | --- | --- |
| A01 | NextDenovo | 2.83 | **36.84/24** | **27.53/29** | **381** | **27.63** | **97.92** | **37.97** |
|  | Flye | 2.84 | 25.15/35 | 25.15/36 | 561 | 27.49 | 97.65 | 35.52 |
| Han | NextDenovo | 2.83 | **41.56/23** | **31.22/28** | **351** | **28.50** | **98.18** | **38.16** |
|  | Flye | 2.84 | 25.1/33 | 24.23/36 | 492 | 28.38 | 97.79 | 34.88 |
| Iran | NextDenovo | 2.83 | **33.31/25** | **31.89/28** | **394** | **28.60** | **98.22** | **42.47** |
|  | Flye | 2.84 | 25.65/34 | 25.57/35 | 665 | 28.44 | 98.09 | 37.13 |
| Ba | NextDenovo | 2.82 | **34.23/25** | **27.79/28** | **349** | **28.33** | **97.99** | **39.06** |
|  | Flye | 2.83 | 24.28/35 | 22.97/39 | 545 | 28.17 | 97.88 | 32.75 |
| SA1 | NextDenovo | 2.83 | **33.34/24** | **28.76/28** | **390** | **28.55** | **98.38** | **40.60** |
|  | Flye | 2.84 | 25.75/34 | 25.46/35 | 720 | 28.32 | 98.23 | 35.65 |
| A02 | NextDenovo | 2.81 | **33.34/26** | **26.72/31** | **389** | **28.49** | **97.91** | **38.16** |
|  | Flye | 2.82 | 25.11/35 | 22.96/38 | 503 | 28.36 | 97.79 | 32.43 |
| A03 | NextDenovo | 2.83 | **31.98/27** | **26.98/32** | **407** | **27.60** | **97.90** | **40.03** |
|  | Flye | 2.84 | 25.67/35 | 24.27/38 | 491 | 27.56 | 97.59 | 34.94 |
| A04 | NextDenovo | 2.83 | **30.81/27** | **26.5/29** | **359** | **27.67** | **97.85** | **36.94** |
|  | Flye | 2.83 | 24.2/37 | 23.47/39 | 488 | 27.60 | 97.70 | 32.82 |
| A05 | NextDenovo | 2.83 | **33.23/25** | **26.88/32** | **399** | **27.61** | **97.86** | **37.39** |
|  | Flye | 2.84 | 25.44/34 | 23.86/38 | 510 | 27.55 | 97.69 | 32.37 |
| A06 | NextDenovo | 2.82 | **28.47/27** | **25.95/32** | **364** | 27.61 | **97.71** | **37.26** |
|  | Flye | 2.83 | 25.08/33 | 24.62/36 | 417 | **27.66** | 97.51 | 32.18 |
| A07 | NextDenovo | 2.83 | **39.5/24** | **30.46/29** | **428** | **27.61** | **97.98** | **39.51** |
|  | Flye | 2.84 | 28.04/30 | 26.2/33 | 653 | 27.51 | 97.88 | 35.59 |
| A08 | NextDenovo | 2.83 | **40.01/22** | **31.24/26** | **421** | **27.68** | **98.07** | **41.76** |
|  | Flye | 2.84 | 28.86/31 | 26.1/33 | 592 | 27.59 | 97.84 | 34.23 |
| A09 | NextDenovo | 2.83 | **35.57/25** | **26.79/30** | **434** | **27.66** | **97.88** | **42.47** |
|  | Flye | 2.84 | 28.19/31 | 26.13/32 | 586 | 27.47 | 97.80 | 37.13 |
| A10 | NextDenovo | 2.83 | **32.59/26** | **28.24/30** | **403** | **27.81** | **98.06** | **41.83** |
|  | Flye | 2.83 | 25.68/34 | 25.08/36 | 571 | 27.72 | 97.74 | 33.85 |
| A11 | NextDenovo | 2.84 | **33.33/28** | **29.38/32** | **448** | **27.60** | **98.15** | **45.17** |
|  | Flye | 2.84 | 27.38/33 | 26.3/33 | 707 | 27.45 | 97.90 | 37.58 |
| A12 | NextDenovo | 2.83 | **37.72/23** | **28.88/30** | **383** | 28.44 | **98.02** | **35.84** |
|  | Flye | 2.83 | 26.04/32 | 25.6/33 | 477 | **28.50** | 98.01 | 30.95 |
| A15 | NextDenovo | 2.81 | **33.07/25** | **26.36/34** | **388** | **27.65** | **97.44** | **36.55** |
|  | Flye | 2.81 | 25.63/33 | 25.08/37 | 527 | 27.56 | 97.26 | 30.24 |
| E1 | NextDenovo | 2.83 | **33.05/26** | **29.18/29** | **374** | **28.65** | **98.10** | **39.58** |
|  | Flye | 2.84 | 25.65/34 | 23.51/35 | 573 | 28.48 | 98.05 | 34.36 |
| Kenya | NextDenovo | 2.83 | **33.12/26** | **25.74/31** | **424** | **27.66** | **97.82** | **36.36** |
|  | Flye | 2.83 | 23.26/34 | 20.11/38 | 538 | 27.58 | 97.57 | 35.46 |
| LX | NextDenovo | 2.82 | **33.13/24** | **27.93/29** | **338** | **28.49** | **98.04** | **42.08** |
|  | Flye | 2.83 | 25.22/32 | 25.05/34 | 528 | 28.37 | 97.83 | 33.33 |
| Mos | NextDenovo | 2.82 | **33.31/24** | **30.26/28** | **336** | **28.42** | **97.96** | **39.25** |
|  | Flye | 2.84 | 25.14/36 | 25.05/38 | 625 | 28.26 | 97.79 | 31.98 |
| Nigeria | NextDenovo | 2.82 | **39.26/23** | **28.52/30** | **374** | 27.62 | **97.58** | **38.35** |
|  | Flye | 2.83 | 25.21/34 | 24.64/37 | 444 | **27.63** | 97.56 | 32.43 |
| Xinjiang | NextDenovo | 2.84 | **33.46/26** | **28.89/29** | 468 | **28.51** | **98.46** | **44.92** |
|  | Flye | 2.84 | 26.89/33 | 26.7/36 | **457** | 28.42 | 98.08 | 38.29 |
| Y10 | NextDenovo | 2.84 | **33.33/25** | **30.48/28** | **391** | **28.33** | **98.04** | **44.34** |
|  | Flye | 2.84 | 25.75/31 | 25.13/33 | 762 | 28.21 | 97.88 | 33.91 |
| Y1 | NextDenovo | 2.83 | **39.63/24** | **31.35/28** | **426** | **28.38** | **98.26** | **43.18** |
|  | Flye | 2.84 | 26.84/31 | 26.46/33 | 594 | 28.34 | 97.99 | 38.16 |
| Y2 | NextDenovo | 2.83 | **40.71/22** | **29.06/29** | **351** | **28.53** | **98.35** | **43.56** |
|  | Flye | 2.84 | 28.25/29 | 26.71/31 | 569 | 28.35 | 98.01 | 38.22 |
| Y3 | NextDenovo | 2.82 | **36.93/25** | **28.55/30** | **402** | **28.56** | 97.96 | **38.22** |
|  | Flye | 2.83 | 24.7/35 | 24.22/36 | 511 | 28.50 | **98.00** | 34.49 |
| Y4 | NextDenovo | 2.82 | **34.86/23** | **29.39/30** | 374 | 28.36 | **97.63** | **34.56** |
|  | Flye | 2.82 | 18.57/41 | 18.23/44 | **373** | **28.45** | 97.34 | 22.33 |
| Y5 | NextDenovo | 2.83 | **33.29/24** | **29.17/28** | **421** | **28.39** | **98.14** | **39.45** |
|  | Flye | 2.83 | 26.66/31 | 26.2/33 | 581 | 28.24 | 98.03 | 33.53 |
| Y6 | NextDenovo | 2.82 | **37.58/25** | **31.97/29** | **384** | **28.45** | **98.16** | **38.74** |
|  | Flye | 2.83 | 28.27/31 | 27.97/33 | 555 | 28.27 | 98.06 | 31.34 |
| Y7 | NextDenovo | 2.83 | **33.32/23** | **31.2/28** | **390** | **28.43** | **98.35** | **46.01** |
|  | Flye | 2.84 | 25.3/33 | 25.1/36 | 553 | 28.36 | 98.01 | 36.74 |
| Y8 | NextDenovo | 2.81 | **36.82/23** | **33.02/26** | **322** | **28.77** | **98.12** | **39.64** |
|  | Flye | 2.82 | 29.1/32 | 28.16/33 | 522 | 28.51 | 97.82 | 34.75 |
| Y9 | NextDenovo | 2.82 | **26.93/31** | **25.75/32** | **362** | **28.30** | 97.26 | **36.42** |
|  | Flye | 2.84 | 24.16/34 | 23.66/35 | 525 | 28.25 | **97.40** | 31.98 |
| Yin | NextDenovo | 2.82 | **33.0/28** | **27.89/31** | **370** | **28.52** | 97.83 | **33.33** |
|  | Flye | 2.83 | 25.12/34 | 24.59/36 | 578 | 28.35 | **97.97** | 32.95 |
| Zang | NextDenovo | 2.82 | **37.23/23** | **31.14/30** | **392** | **28.43** | **97.98** | **36.87** |
|  | Flye | 2.83 | 26.52/32 | 26.4/35 | 545 | 28.21 | 97.93 | 33.01 |
| Average | NextDenovo | 2.83 | **34.80/25** | **28.89/30** | **388** | **28.17** | **97.99** | **39.60** |
|  | Flye | 2.83 | 25.77/33 | 24.89/36 | 553 | 28.06 | 97.82 | 33.93 |

NG50 is the length N that 50% of the reference genome is covered in contigs with length ≥ N. LG50 is the number of contigs with length ≥ NG50. NGA50 is NG50 of aligned blocks that are obtained by breaking contigs at misassembly events and removing all unaligned bases. LGA50 is the number of aligned blocks with length ≥ NGA50. Misassemblies and QV are evaluated by QUAST, where QV is defined as $-10\times\log_{10} (\frac{\# mismatches per 100 kbp + \# indels per 100 kbp}{100 kbp})$. Gene completeness and “multicopy genes retained” are reported by asmgene, “multicopy genes retained” is the percentage of multicopy genes in the reference genome that remain multicopy genes in the assembly. QV, Gene completeness and “multicopy genes retained” wereevaluated using the polished assemblies and other metrics were evaluated using the raw assemblies. Best results for each metric are highlighted in bold.

**Table S8: Gene completeness of 35 human assemblies**

| **Sample** | **Software** | **Full_sgl** | **Full_dup** | **Frag** | **Dup_cnt** | **Dup_sum** |
| --- | --- | --- | --- | --- | --- | --- |
| CHM13 | **-** | 36,447 | 0 | 0 | 1,554 | 4,741 |
| A01 | NextDenovo | 35,644 | 45 | 19 | 590 | 2,483 |
|  | Flye | 35,545 | 46 | 29 | 552 | 2,247 |
| Han | NextDenovo | 35,727 | 56 | 15 | 593 | 2,478 |
|  | Flye | 35,593 | 50 | 24 | 542 | 2,173 |
| Iran | NextDenovo | 35,745 | 52 | 17 | 660 | 2,555 |
|  | Flye | 35,694 | 57 | 31 | 577 | 2,299 |
| Ba | NextDenovo | 35,657 | 59 | 13 | 607 | 2,363 |
|  | Flye | 35,622 | 51 | 34 | 509 | 2,152 |
| SA1 | NextDenovo | 35,802 | 55 | 11 | 631 | 2,557 |
|  | Flye | 35,755 | 47 | 32 | 554 | 2,257 |
| A02 | NextDenovo | 35,627 | 59 | 10 | 593 | 2,279 |
|  | Flye | 35,590 | 52 | 32 | 504 | 2,041 |
| A03 | NextDenovo | 35,618 | 63 | 15 | 622 | 2,547 |
|  | Flye | 35,516 | 51 | 40 | 543 | 2,197 |
| A04 | NextDenovo | 35,600 | 65 | 12 | 574 | 2,420 |
|  | Flye | 35,548 | 59 | 32 | 510 | 2,117 |
| A05 | NextDenovo | 35,613 | 55 | 17 | 581 | 2,356 |
|  | Flye | 35,545 | 60 | 35 | 503 | 2,070 |
| A06 | NextDenovo | 35,550 | 62 | 15 | 579 | 2,438 |
|  | Flye | 35,491 | 50 | 39 | 500 | 2,130 |
| A07 | NextDenovo | 35,660 | 49 | 7 | 614 | 2,499 |
|  | Flye | 35,641 | 34 | 30 | 553 | 2,232 |
| A08 | NextDenovo | 35,684 | 60 | 10 | 649 | 2,584 |
|  | Flye | 35,612 | 49 | 30 | 532 | 2,194 |
| A09 | NextDenovo | 35,606 | 70 | 10 | 660 | 2,651 |
|  | Flye | 35,594 | 52 | 31 | 577 | 2,326 |
| A10 | NextDenovo | 35,672 | 69 | 14 | 650 | 2,652 |
|  | Flye | 35,561 | 61 | 27 | 526 | 2,205 |
| A11 | NextDenovo | 35,701 | 72 | 12 | 702 | 2,707 |
|  | Flye | 35,626 | 55 | 34 | 584 | 2,270 |
| A12 | NextDenovo | 35,693 | 34 | 14 | 557 | 2,369 |
|  | Flye | 35,675 | 47 | 39 | 481 | 2,072 |
| A15 | NextDenovo | 35,461 | 53 | 15 | 568 | 2,228 |
|  | Flye | 35,390 | 59 | 39 | 470 | 1,901 |
| E1 | NextDenovo | 35,704 | 49 | 13 | 615 | 2,505 |
|  | Flye | 35,693 | 43 | 45 | 534 | 2,220 |
| Kenya | NextDenovo | 35,582 | 69 | 17 | 565 | 2,413 |
|  | Flye | 35,506 | 54 | 31 | 551 | 2,151 |
| LX | NextDenovo | 35,676 | 57 | 14 | 654 | 2,552 |
|  | Flye | 35,607 | 49 | 22 | 518 | 2,183 |
| Mos | NextDenovo | 35,649 | 53 | 16 | 610 | 2,471 |
|  | Flye | 35,593 | 49 | 34 | 497 | 2,169 |
| Nigeria | NextDenovo | 35,506 | 58 | 13 | 596 | 2,388 |
|  | Flye | 35,502 | 56 | 28 | 504 | 2,110 |
| Xinjiang | NextDenovo | 35,821 | 66 | 10 | 698 | 2,647 |
|  | Flye | 35,685 | 63 | 33 | 595 | 2,298 |
| Y10 | NextDenovo | 35,687 | 47 | 6 | 689 | 2,638 |
|  | Flye | 35,636 | 39 | 30 | 527 | 2,163 |
| Y1 | NextDenovo | 35,759 | 53 | 10 | 671 | 2,641 |
|  | Flye | 35,662 | 53 | 37 | 593 | 2,309 |
| Y2 | NextDenovo | 35,788 | 56 | 7 | 677 | 2,652 |
|  | Flye | 35,669 | 53 | 35 | 594 | 2,385 |
| Y3 | NextDenovo | 35,654 | 50 | 16 | 594 | 2,396 |
|  | Flye | 35,674 | 43 | 37 | 536 | 2,187 |
| Y4 | NextDenovo | 35,530 | 53 | 16 | 537 | 2,279 |
|  | Flye | 35,430 | 48 | 63 | 347 | 1,799 |
| Y5 | NextDenovo | 35,710 | 58 | 13 | 613 | 2,454 |
|  | Flye | 35,688 | 41 | 37 | 521 | 2,139 |
| Y6 | NextDenovo | 35,729 | 49 | 12 | 602 | 2,462 |
|  | Flye | 35,693 | 47 | 32 | 487 | 2,082 |
| Y7 | NextDenovo | 35,783 | 62 | 8 | 715 | 2,703 |
|  | Flye | 35,671 | 50 | 42 | 571 | 2,241 |
| Y8 | NextDenovo | 35,692 | 69 | 9 | 616 | 2,363 |
|  | Flye | 35,613 | 38 | 31 | 540 | 2,017 |
| Y9 | NextDenovo | 35,397 | 50 | 13 | 566 | 2,338 |
|  | Flye | 35,460 | 40 | 38 | 497 | 2,069 |
| Yin | NextDenovo | 35,611 | 46 | 12 | 518 | 2,211 |
|  | Flye | 35,664 | 44 | 42 | 512 | 2,140 |
| Zang | NextDenovo | 35,671 | 41 | 10 | 573 | 2,336 |
|  | Flye | 35,652 | 39 | 28 | 513 | 2,155 |
| Average | NextDenovo | 35,657 | 56 | 13 | 615 | 2,475 |
|  | Flye | 35,626 | 48 | 33 | 556 | 2,234 |

Reported by paftools (v2.24) asmgene function using the polished assemblies. Full_sgl is the count of single copy genes in the reference genome that remain single copy genes in the assembly. Full_dup is the count of single copy genes in the reference genome that remain multi-copy genes in the assembly. Frag is the count of single copy genes in the reference genome that remain fragments in the assembly. Dup_cnt is the count of multi-copy genes in the reference genome that remain multi-copy genes in the assembly. Dup_sum is the count of multi-copy genes in the assembly that are also multi-copy genes in the reference genome.

**Table S9: Summary statistic of segmental duplications across 35 human assemblies**

| No. | Sample | Country | Population | Super-population | SDs Length (Mbp) | Percentage of SDs (%) | Filter SDs Length (Mbp) | Percentage of filter SDs (%) | New SDs Length (Mbp) | Percentage of new SDs (%) |
| --- | --- | --- | --- | --- | --- | --- | --- | --- | --- | --- |
| 1 | A01 | Benin | AFR | AFR | 195.79 | 6.89 | 134.59 | 4.73 | 43.72 | 22.33 |
| 2 | A03 | Mali | AFR | AFR | 193.49 | 6.81 | 134.00 | 4.71 | 46.16 | 23.86 |
| 3 | A04 | Tanzania | AFR | AFR | 189.62 | 6.68 | 130.16 | 4.59 | 44.24 | 23.33 |
| 4 | A05 | Cameroon | AFR | AFR | 192.48 | 6.77 | 132.77 | 4.67 | 44.46 | 23.10 |
| 5 | A06 | Congo | AFR | AFR | 186.50 | 6.58 | 128.02 | 4.51 | 44.72 | 23.98 |
| 6 | A07 | Mali | AFR | AFR | 198.81 | 6.99 | 137.36 | 4.83 | 49.23 | 24.76 |
| 7 | A08 | Rwanda | AFR | AFR | 200.49 | 7.04 | 139.95 | 4.91 | 49.59 | 24.74 |
| 8 | A09 | Togo | AFR | AFR | 200.34 | 7.04 | 138.89 | 4.88 | 48.07 | 23.99 |
| 9 | A10 | N.Sudan | AFR | AFR | 198.00 | 6.96 | 137.76 | 4.84 | 48.35 | 24.42 |
| 10 | A11 | Zambia | AFR | AFR | 207.47 | 7.27 | 145.85 | 5.11 | 52.19 | 25.15 |
| 11 | A15 | Zimbabwe | AFR | AFR | 171.80 | 6.09 | 118.42 | 4.20 | 43.05 | 25.06 |
| 12 | Nigeria | Nigeria | AFR | AFR | 188.19 | 6.64 | 129.58 | 4.57 | 40.33 | 21.43 |
| 13 | Kenya | Kenya | AFR | AFR | 189.99 | 6.69 | 130.41 | 4.59 | 43.23 | 22.76 |
| 14 | Han | China | EA | EA | 194.82 | 6.85 | 134.76 | 4.74 | 43.09 | 22.12 |
| 15 | LX | China | EA | EA | 189.20 | 6.67 | 129.40 | 4.56 | 41.58 | 21.98 |
| 16 | Xinjiang | China | EA | EA | 202.68 | 7.11 | 142.60 | 5.00 | 49.90 | 24.62 |
| 17 | Zang | China | EA | EA | 192.03 | 6.76 | 134.59 | 4.74 | 43.15 | 22.47 |
| 18 | Mos | China | EA | EA | 189.59 | 6.68 | 129.78 | 4.57 | 41.97 | 22.14 |
| 19 | Y10 | Japan | EA | EA | 203.57 | 7.14 | 143.45 | 5.03 | 48.72 | 23.93 |
| 20 | Iran | Iran | SA | SA | 194.60 | 6.85 | 136.33 | 4.80 | 43.93 | 22.57 |
| 21 | Yin | India | SA | SA | 184.20 | 6.51 | 123.66 | 4.37 | 39.24 | 21.30 |
| 22 | Y9 | India/NE | SA | SA | 187.38 | 6.61 | 128.28 | 4.52 | 43.22 | 23.07 |
| 23 | Y5 | Nepal | SA | SA | 196.73 | 6.92 | 138.13 | 4.86 | 49.58 | 25.20 |
| 24 | A12 | Bangladesh | SA | SA | 191.37 | 6.74 | 132.02 | 4.65 | 43.30 | 22.63 |
| 25 | A02 | Siri Lanka | SA | SA | 178.43 | 6.31 | 122.51 | 4.33 | 43.20 | 24.21 |
| 26 | Y3 | Pakistan | SA | SA | 190.54 | 6.71 | 130.50 | 4.60 | 43.14 | 22.64 |
| 27 | Y4 | Laos | SEA | SEA | 185.21 | 6.54 | 125.37 | 4.43 | 40.73 | 21.99 |
| 28 | Y1 | Indonesia | SEA | SEA | 198.11 | 6.96 | 139.16 | 4.89 | 47.96 | 24.21 |
| 29 | Y6 | Vietnam | SEA | SEA | 194.20 | 6.84 | 134.19 | 4.72 | 41.35 | 21.29 |
| 30 | Y7 | Thailand | SEA | SEA | 201.27 | 7.07 | 139.49 | 4.90 | 48.61 | 24.15 |
| 31 | E1 | France | EUR | EUR | 191.86 | 6.76 | 133.94 | 4.72 | 46.65 | 24.32 |
| 32 | Y8 | Germany | EUR | EUR | 184.55 | 6.51 | 128.32 | 4.53 | 41.43 | 22.45 |
| 33 | Y2 | Yemen | WA | WA | 203.66 | 7.15 | 143.17 | 5.03 | 47.20 | 23.18 |
| 34 | Ba | Papua New Guinea | SWP | SWP | 187.45 | 6.61 | 128.56 | 4.53 | 42.78 | 22.82 |
| 35 | SA1 | Peru | AMR | AMR | 200.36 | 7.04 | 140.28 | 4.93 | 44.46 | 22.19 |

**Table S10: Duplicate gene annotation across 35 human assemblies. (Table is huge. Please refer to the additional file 2)**

**Table S11: Statistics of assemblies with HiFi data**

| **Sample** | **Software** | **Assembly size (Mb)** | **NG50 (Mb)/LG50** | **NGA50 (Mb)/LGA50** | **No. of misassemblies** | **QV** | **Gene completeness (%)** | **Wall clock time (hour)** | **Cpu time (hour)** | **Maximum RAM (GB)** |
| --- | --- | --- | --- | --- | --- | --- | --- | --- | --- | --- |
| *A. thaliana* (183X) | NextDenovo | 121.11 | 8.51/6 | - | - | - | 99.10 | 6.15 | 312.44 | 9.67 |
|  | Hifiasm | 192.96 | **13.41/4** | - | - | - | **99.20** | **4.73** | 240.24 | 44.06 |
| *D. melanogaster* (38X) | NextDenovo | 136.63 | 3.86/11 | 3.40/12 | **495** | 24.35 | **98.80** | 0.35 | 22.50 | 6.85 |
|  | Hifiasm | 154.98 | **21.50/3** | **16.71/4** | 1,308 | **24.46** | 98.60 | **0.19** | 13.23 | 18.73 |
| *O. sativa* (50X) | NextDenovo | 393.31 | 6.54/21 | - | - | - | **98.70** | 2.27 | 101.92 | 10.36 |
|  | Hifiasm | 414.89 | **26.95/6** | - | - | - | **98.70** | **1.77** | 85.26 | 23.32 |
| *H. sapiens* (CHM13, 33X) | NextDenovo | 2,854.94 | 31.41/27 | 30.44/28 | 139 | 39.55 | 99.12 | 22.75 | 1112.61 | 30.20 |
|  | Hifiasm | 2,960.54 | **96.74/11** | **87.68/13** | **60** | **47.72** | **100.00** | **6.68** | 307.02 | 112.42 |

NG50 is the length N that 50% of the reference genome is covered in contigs with length ≥ N. LG50 is the number of contigs with length ≥ NG50. NGA50 is an NG50 of aligned blocks that are obtained by breaking contigs at misassembly events and removing all unaligned bases. LGA50 is the number of aligned blocks with length ≥ NGA50. Misassemblies and QV are evaluated by QUAST, where QV is defined as $-10\times\log_{10} (\frac{\# mismatches per 100 kbp + \# indels per 100 kbp}{100 kbp})$. Gene completeness of non-human assemblies is represented by the complete BUSCO value; Gene completeness of human assemblies is reported by asmgene. Since the reference sequences of *A. thaliana* and *O. sativa* were assembled with Hifiasm, we were unable to evaluate some metrics. All the software was tested on the same computer with 60 CPUs and 504 GB RAM of memory. Best results for each metric are highlighted in bold.
