# Supplementary material for: NextDenovo: an efficient error correction and accurate assembly tool for noisy long reads
Source: Genome Biol. 2024 Apr 26;25:107. doi: 10.1186/s13059-024-03252-4 (PMC11046930; doi:10.1186/s13059-024-03252-4)
Supplement: Supplementary file 3 — Additional file 3. Software commands used in this study. [file 13059_2024_3252_MOESM3_ESM.docx]

## Software commands

#### Error correction

###### Simulation data

**Simulate NanoPore data:**

python NanoSim/src/simulator.py genome -rg chr01.fa -c NanoSim/pre-trained_models/human_NA12878_DNA_FAB49712_guppy/training -n 1631727 -b guppy

cat simulated_aligned_reads.fasta simulated_unaligned_reads.fasta > ont.simulation.fa

**Run canu to correct reads:**

canu -correct useGrid=false -p sim_ont -d asm genomeSize=248956422 corMemory=24g -nanopore-raw ont.simulation.fa.gz

**Run NextDenovo to correct reads:**

run.cfg:

[General]

job_type = local # local, slurm, sge, pbs, lsf

job_prefix = nextDenovo

task = correct # all, correct, assemble

rewrite = yes # yes/no

deltmp = yes

parallel_jobs = 4 # number of tasks used to run in parallel

read_type = ont

input_fofn = input.fofn

workdir = 01_rundir

[correct_option]

read_cutoff = 1k

seed_cutoff = 12089 # minimum seed length

pa_correction = 2 # number of corrected tasks used to run in parallel, overwrite ${parallel_jobs} only for this step

seed_cutfiles = 4

sort_options = -m 40g -t 8 -k 40 # -k, max depth of each overlap, should <= average sequencing depth

minimap2_options_raw = -x ava-ont -t 8 # change to ava-pb for PacBio CLR data

correction_options = -p 16 –b

command:

nextDenovo run.cfg

**Run Necat to correct reads:**

run.cfg:

PROJECT=chr1

ONT_READ_LIST=input.fofn

GENOME_SIZE=248956422

THREADS=32

MIN_READ_LENGTH=1000

PREP_OUTPUT_COVERAGE=50.32

OVLP_FAST_OPTIONS=-n 500 -z 20 -b 2000 -e 0.5 -j 0 -u 1 -a 1000

OVLP_SENSITIVE_OPTIONS=-n 500 -z 10 -e 0.5 -j 0 -u 1 -a 1000

CNS_FAST_OPTIONS=-a 2000 -x 4 -y 12 -l 1000 -e 0.5 -p 0.8 -u 0

CNS_SENSITIVE_OPTIONS=-a 2000 -x 4 -y 12 -l 1000 -e 0.5 -p 0.8 -u 0

TRIM_OVLP_OPTIONS=-n 100 -z 10 -b 2000 -e 0.5 -j 1 -u 1 -a 400

ASM_OVLP_OPTIONS=-n 100 -z 10 -b 2000 -e 0.5 -j 1 -u 0 -a 400

NUM_ITER=2

CNS_OUTPUT_COVERAGE=50.32

CLEANUP=1

USE_GRID=false

GRID_NODE=0

GRID_OPTIONS=

SMALL_MEMORY=0

FSA_OL_FILTER_OPTIONS=

FSA_ASSEMBLE_OPTIONS=

FSA_CTG_BRIDGE_OPTIONS=

POLISH_CONTIGS=true

command:

perl necat.pl correct run.cfg

**Run Consent to correct reads:**

CONSENT-correct --nproc 32 --in ont.simulation.fa --out result.fasta --type ONT

###### Actual biological data

**Run canu to correct reads:**

canu -correct useGrid=false -p chr1_ont -d asm genomeSize=248956422 -nanopore-raw chr1.id.fra0.65.fa

**Run NextDenovo to correct reads:**

run.cfg:

[General]

job_type = local # local, slurm, sge, pbs, lsf

job_prefix = nextDenovo

task = correct # all, correct, assemble

rewrite = yes # yes/no

deltmp = yes

parallel_jobs = 4 # number of tasks used to run in parallel

read_type = ont

input_fofn = input.fofn

workdir = 01_rundir

[correct_option]

read_cutoff = 1k

seed_cutoff = 46726 # minimum seed length

pa_correction = 2 # number of corrected tasks used to run in parallel, overwrite ${parallel_jobs} only for this step

seed_cutfiles = 4

sort_options = -m 40g -t 8 -k 40 # -k, max depth of each overlap, should <= average sequencing depth

minimap2_options_raw = -x ava-ont -k 17 -t 8 # change to ava-pb for PacBio CLR data

correction_options = -p 16 -b

command:

nextDenovo run.cfg

**Run Necat to correct reads:**

run.cfg:

PROJECT=chr1

ONT_READ_LIST=input.fofn

GENOME_SIZE=248956422

THREADS=32

MIN_READ_LENGTH=1000

PREP_OUTPUT_COVERAGE=42.38

OVLP_FAST_OPTIONS=-n 500 -z 20 -b 2000 -e 0.5 -j 0 -u 1 -a 1000

OVLP_SENSITIVE_OPTIONS=-n 500 -z 10 -e 0.5 -j 0 -u 1 -a 1000

CNS_FAST_OPTIONS=-a 2000 -x 4 -y 12 -l 1000 -e 0.5 -p 0.8 -u 0

CNS_SENSITIVE_OPTIONS=-a 2000 -x 4 -y 12 -l 1000 -e 0.5 -p 0.8 -u 0

TRIM_OVLP_OPTIONS=-n 100 -z 10 -b 2000 -e 0.5 -j 1 -u 1 -a 400

ASM_OVLP_OPTIONS=-n 100 -z 10 -b 2000 -e 0.5 -j 1 -u 0 -a 400

NUM_ITER=2

CNS_OUTPUT_COVERAGE=42.38

CLEANUP=1

USE_GRID=false

GRID_NODE=0

GRID_OPTIONS=

SMALL_MEMORY=0

FSA_OL_FILTER_OPTIONS=

FSA_ASSEMBLE_OPTIONS=

FSA_CTG_BRIDGE_OPTIONS=

POLISH_CONTIGS=true

command:

perl necat.pl correct run.cfg

**Run Consent to correct reads:**

CONSENT-correct --nproc 32 --in chr1.id.fra0.65.fa --out result.fasta --type ONT

###### Evaluation

**Map reads to reference:**

minimap2 -t 10 -x map-ont –c chr01.fa corrected.fasta > corrected.fasta.paf

**Caculate the average error rate of reads:**

ls *.paf|while read line;do echo $line && grep "tp:A:P" $line|awk '($4-$3)/$2>=0.8'|awk '{x+=$10/$11}END{print 1-x/NR}';done;

**Caculate the count of reads mapped with ≥99% coverage:**

ls *.paf|while read line;do echo $line && awk '($4-$3)/$2>=0.99' $line|cut -f 1 |sort|uniq|wc -l;done;

**Caculate the count of reads mapped with ≥97% identity:**

ls *.paf|while read line;do echo $line && awk '$10/$11>=0.97' $line|cut -f 1 |sort|uniq|wc -l;done;

**Caculate the count of reads with chimeric alignments:**

ls *.paf|while read line;do echo $line && grep "tp:A:P" $line|cut -f 1|sort|uniq -d|wc -l;done;

#### Contig assembly

###### *Arabidopsis thaliana*

**Run Canu to assemble:**

canu useGrid=false -p asm -d asm genomeSize=125m -nanopore-raw CRR302667.fastq.gz

**Run Flye to assemble:**

flye --nano-raw CRR302667.fastq.gz --out-dir asm1 --threads 60 -g 125m

**Run NextDenovo to assemble:**

run.cfg:

[General]

job_type = local # local, slurm, sge, pbs, lsf

job_prefix = nextDenovo

task = all # all, correct, assemble

rewrite = yes # yes/no

deltmp = yes

parallel_jobs = 10 # number of tasks used to run in parallel

input_type = raw # raw, corrected

read_type = ont # clr, ont, hifi

input_fofn = input.fofn1

workdir = 01_rundir

[correct_option]

read_cutoff = 1k

genome_size = 125m # estimated genome size

sort_options = -m 400g -t 15

minimap2_options_raw = -t 6

pa_correction = 4 # number of corrected tasks used to run in parallel, each corrected task requires ~TOTAL_INPUT_BASES/4 bytes of memory usage.

correction_options = -p 15

[assemble_option]

minimap2_options_cns = -t 6 --wn 21 --kn 21

nextgraph_options = -a 1

command:

nextDenovo run.cfg

**Run Necat to assemble:**

run.cfg:

PROJECT=alta

ONT_READ_LIST=input.fofn

GENOME_SIZE=125000000

THREADS=60

MIN_READ_LENGTH=1000

PREP_OUTPUT_COVERAGE=40

OVLP_FAST_OPTIONS=-n 500 -z 20 -b 2000 -e 0.5 -j 0 -u 1 -a 1000

OVLP_SENSITIVE_OPTIONS=-n 500 -z 10 -e 0.5 -j 0 -u 1 -a 1000

CNS_FAST_OPTIONS=-a 2000 -x 4 -y 12 -l 1000 -e 0.5 -p 0.8 -u 0

CNS_SENSITIVE_OPTIONS=-a 2000 -x 4 -y 12 -l 1000 -e 0.5 -p 0.8 -u 0

TRIM_OVLP_OPTIONS=-n 100 -z 10 -b 2000 -e 0.5 -j 1 -u 1 -a 400

ASM_OVLP_OPTIONS=-n 100 -z 10 -b 2000 -e 0.5 -j 1 -u 0 -a 400

NUM_ITER=2

CNS_OUTPUT_COVERAGE=30

CLEANUP=1

USE_GRID=false

GRID_NODE=0

GRID_OPTIONS=

SMALL_MEMORY=0

FSA_OL_FILTER_OPTIONS=

FSA_ASSEMBLE_OPTIONS=

FSA_CTG_BRIDGE_OPTIONS=

POLISH_CONTIGS=true

command:

perl necat.pl correct run.cfg

perl necat.pl assemble run.cfg

perl necat.pl bridge run.cfg

**Run Wtdbg2 to assemble:**

wtdbg2.pl -xont -g125m -t 60 -o wtdbg2.asm /project2/huj/01_alta_ont/CRR302667.fastq.gz

###### *Drosophila melanogaster*

**Run Canu to assemble:**

canu useGrid=false -p asm -d asm genomeSize=144m -nanopore-raw SRR6702603.fasta.gz SRR6821890.fasta.gz

**Run Flye to assemble:**

flye --nano-raw SRR6702603.fasta.gz SRR6821890.fasta.gz --out-dir asm --threads 60 -g 144m

**Run NextDenovo to assemble:**

run.cfg:

[General]

job_type = local # local, slurm, sge, pbs, lsf

job_prefix = nextDenovo

task = all # all, correct, assemble

rewrite = yes # yes/no

deltmp = yes

parallel_jobs = 10 # number of tasks used to run in parallel

input_type = raw # raw, corrected

read_type = ont # clr, ont, hifi

input_fofn = input.fofn

workdir = 01_rundir

[correct_option]

read_cutoff = 1k

genome_size = 144m # estimated genome size

sort_options = -m 400g -t 15

minimap2_options_raw = -t 6

pa_correction = 4 # number of corrected tasks used to run in parallel, each corrected task requires ~TOTAL_INPUT_BASES/4 bytes of memory usage.

correction_options = -p 15

[assemble_option]

minimap2_options_cns = -t 6

nextgraph_options = -a 1

command:

nextDenovo run.cfg

**Run Necat to assemble:**

run.cfg:

PROJECT=dmel

ONT_READ_LIST=input.fofn

GENOME_SIZE=144000000

THREADS=60

MIN_READ_LENGTH=1000

PREP_OUTPUT_COVERAGE=40

OVLP_FAST_OPTIONS=-n 500 -z 20 -b 2000 -e 0.5 -j 0 -u 1 -a 1000

OVLP_SENSITIVE_OPTIONS=-n 500 -z 10 -e 0.5 -j 0 -u 1 -a 1000

CNS_FAST_OPTIONS=-a 2000 -x 4 -y 12 -l 1000 -e 0.5 -p 0.8 -u 0

CNS_SENSITIVE_OPTIONS=-a 2000 -x 4 -y 12 -l 1000 -e 0.5 -p 0.8 -u 0

TRIM_OVLP_OPTIONS=-n 100 -z 10 -b 2000 -e 0.5 -j 1 -u 1 -a 400

ASM_OVLP_OPTIONS=-n 100 -z 10 -b 2000 -e 0.5 -j 1 -u 0 -a 400

NUM_ITER=2

CNS_OUTPUT_COVERAGE=30

CLEANUP=1

USE_GRID=false

GRID_NODE=0

GRID_OPTIONS=

SMALL_MEMORY=0

FSA_OL_FILTER_OPTIONS=

FSA_ASSEMBLE_OPTIONS=

FSA_CTG_BRIDGE_OPTIONS=

POLISH_CONTIGS=true

command:

perl necat.pl correct run.cfg

perl necat.pl assemble run.cfg

perl necat.pl bridge run.cfg

**Run Wtdbg2 to assemble:**

wtdbg2.pl -xont -g144m -t 60 -o wtdbg2.asm SRR6702603.fasta.gz SRR6821890.fasta.gz

###### *Oryza sativa*

**Run Hifiasm to assemble:**

hifiasm -o rice -t 20 input.hifi.fa

awk '/^S/{print ">"$2;print $3}' rice.p_ctg.gfa > rice.p_ctg.fa

**Run Canu to assemble:**

canu useGrid=false -p asm -d asm genomeSize=400m corMemory=32 -nanopore-raw ont.fq.gz

**Run Flye to assemble:**

flye --nano-raw ont.fq.gz --out-dir asm --threads 60 -g 400m

**Run NextDenovo to assemble:**

run.cfg:

[General]

job_type = local # local, slurm, sge, pbs, lsf

job_prefix = nextDenovo

task = all # all, correct, assemble

rewrite = yes # yes/no

deltmp = yes

parallel_jobs = 10 # number of tasks used to run in parallel

input_type = raw # raw, corrected

read_type = ont # clr, ont, hifi

input_fofn = input.fofn

workdir = 01_rundir

[correct_option]

read_cutoff = 1k

genome_size = 400m # estimated genome size

sort_options = -m 400g -t 15

minimap2_options_raw = -t 6

pa_correction = 4 # number of corrected tasks used to run in parallel, each corrected task requires ~TOTAL_INPUT_BASES/4 bytes of memory usage.

correction_options = -p 15

[assemble_option]

minimap2_options_cns = -t 6

nextgraph_options = -a 1

command:

nextDenovo run.cfg

**Run Necat to assemble:**

run.cfg:

PROJECT=rice

ONT_READ_LIST=input.fofn

GENOME_SIZE=400000000

THREADS=60

MIN_READ_LENGTH=1000

PREP_OUTPUT_COVERAGE=40

OVLP_FAST_OPTIONS=-n 500 -z 20 -b 2000 -e 0.5 -j 0 -u 1 -a 1000

OVLP_SENSITIVE_OPTIONS=-n 500 -z 10 -e 0.5 -j 0 -u 1 -a 1000

CNS_FAST_OPTIONS=-a 2000 -x 4 -y 12 -l 1000 -e 0.5 -p 0.8 -u 0

CNS_SENSITIVE_OPTIONS=-a 2000 -x 4 -y 12 -l 1000 -e 0.5 -p 0.8 -u 0

TRIM_OVLP_OPTIONS=-n 100 -z 10 -b 2000 -e 0.5 -j 1 -u 1 -a 400

ASM_OVLP_OPTIONS=-n 100 -z 10 -b 2000 -e 0.5 -j 1 -u 0 -a 400

NUM_ITER=2

CNS_OUTPUT_COVERAGE=30

CLEANUP=1

USE_GRID=false

GRID_NODE=0

GRID_OPTIONS=

SMALL_MEMORY=0

FSA_OL_FILTER_OPTIONS=

FSA_ASSEMBLE_OPTIONS=

FSA_CTG_BRIDGE_OPTIONS=

POLISH_CONTIGS=true

command:

perl necat.pl correct run.cfg

perl necat.pl assemble run.cfg

perl necat.pl bridge run.cfg

**Run Wtdbg2 to assemble:**

wtdbg2.pl -xont -g400m -t 60 -o wtdbg2.asm ont.fq.gz

###### *Zea mays*

**Run Canu to assemble:**

canu -p asm -d asm genomeSize=2300m -nanopore-raw maize.ont.50x.fasta gridEngineResourceOption="-pe smp THREADS -l vf=MEMORY" gridOptionsovs="-pe smp 5" batOptions="-M 350"

**Run Flye to assemble:**

flye --nano-raw /home/huj/work2/06_maize_v2/maize.ont.50x.fasta --out-dir asm --threads 60 -g 2300m

**Run NextDenovo to assemble:**

run.cfg:

[General]

job_type = sge # local, slurm, sge, pbs, lsf

job_prefix = nextDenovo

task = all # all, correct, assemble

rewrite = yes # yes/no

deltmp = yes

parallel_jobs = 28 # number of tasks used to run in parallel

input_type = raw # raw, corrected

read_type = ont # clr, ont, hifi

input_fofn = input.fofn

workdir = 01_rundir

[correct_option]

read_cutoff = 1k

genome_size = 2300m # estimated genome size

sort_options = -m 200g -t 32

minimap2_options_raw = -t 8

pa_correction = 7 # number of corrected tasks used to run in parallel, each corrected task requires ~TOTAL_INPUT_BASES/4 bytes of memory usage.

correction_options = -p 32

[assemble_option]

minimap2_options_cns = -t 8

nextgraph_options = -a 1

command:

nextDenovo run.cfg

**Run Necat to assemble:**

run.cfg:

PROJECT=maize

ONT_READ_LIST=input.fofn

GENOME_SIZE=2300000000

THREADS=32

MIN_READ_LENGTH=1000

PREP_OUTPUT_COVERAGE=40

OVLP_FAST_OPTIONS=-n 500 -z 20 -b 2000 -e 0.5 -j 0 -u 1 -a 1000

OVLP_SENSITIVE_OPTIONS=-n 500 -z 10 -e 0.5 -j 0 -u 1 -a 1000

CNS_FAST_OPTIONS=-a 2000 -x 4 -y 12 -l 1000 -e 0.5 -p 0.8 -u 0

CNS_SENSITIVE_OPTIONS=-a 2000 -x 4 -y 12 -l 1000 -e 0.5 -p 0.8 -u 0

TRIM_OVLP_OPTIONS=-n 100 -z 10 -b 2000 -e 0.5 -j 1 -u 1 -a 400

ASM_OVLP_OPTIONS=-n 100 -z 10 -b 2000 -e 0.5 -j 1 -u 0 -a 400

NUM_ITER=2

CNS_OUTPUT_COVERAGE=30

CLEANUP=1

USE_GRID=true

GRID_NODE=7

GRID_OPTIONS=

SMALL_MEMORY=0

FSA_OL_FILTER_OPTIONS=

FSA_ASSEMBLE_OPTIONS=

FSA_CTG_BRIDGE_OPTIONS=

POLISH_CONTIGS=true

command:

perl necat.pl correct run.cfg

perl necat.pl assemble run.cfg

perl necat.pl bridge run.cfg

**Run Wtdbg2 to assemble:**

wtdbg2.pl -xont -g 2300m -t 60 -o wtdbg2.asm maize.ont.50x.fasta

###### Human

**Run Flye to assemble:**

python flye --nano-raw sample.fasta.gz -t 56 -g 3g -o flye

**Run NextDenovo to assemble:**

run.cfg:

[General]

job_type = pbs

job_prefix = nextDenovo

task = all # 'all', 'correct', 'assemble'

rewrite = yes # yes/no

deltmp = yes

rerun = 3

parallel_jobs = 40

input_type = raw

read_type = ont

input_fofn = ./input.fofn

workdir = ./01_rundir

[correct_option]

read_cutoff = 1k

genome_size = 3g

pa_correction = 10

sort_options = -m 250g -t 25

minimap2_options_raw = -t 7

correction_options = -p 28

[assemble_option]

minimap2_options_cns = -t 7

nextgraph_options = -a 1

command:

nextDenovo run.cfg

#### Polishing with long and short reads

**Run NextPolish to correct errors in assemblies:**

run.cfg:

[General]

job_type = sge

job_prefix = nextPolish

task = best

rewrite = yes

deltmp = yes

rerun = 3

parallel_jobs = 20

multithread_jobs = 8

genome = ASSEMBLY.fasta #assembly result in fasta format

workdir = ./01_rundir

genome_size = auto

polish_options = -p {multithread_jobs}

[sgs_option] #optional

sgs_fofn = ./sgs.fofn # short reads

sgs_options = -max_depth 100 -bwa

[lgs_option] #optional

lgs_fofn = ./lgs.fofn # long reads

lgs_options = -min_read_len 1k -max_depth 100

lgs_minimap2_options = -x map-ont

command:

nextPolish run.cfg

#### Quast

###### *Arabidopsis thaliana*

quast.py --eukaryote --large --min-identity 80 --threads 20 -r $ref -m 10000 nd.fa canu.fa flye.fa wtdbg.fa necat.fa

###### *Drosophila melanogaster*

quast.py --eukaryote --large --min-identity 80 --threads 20 -r $ref -m 10000 nd.fa canu.fa flye.fa wtdbg.fa necat.fa

###### *Oryza sativa*

quast.py --fragmented --eukaryote --large --min-identity 80 --threads 20 -r $ref -m 10000 nd.fa canu.fa flye.fa wtdbg.fa necat.fa

###### *Zea mays*

quast.py --fragmented --eukaryote --large --min-identity 80 --threads 20 -r $ref -m 10000 nd.fa canu.fa flye.fa wtdbg.fa necat.fa

###### Human

quast.py --eukaryote --large --min-identity 80 --threads 20 -m 50000 -r GCA_009914755.4_CHM13_T2T_v2.0_genomic.fna.gz -o sample.quast nd.asm.fasta assembly.fasta

#### BUSCO for non-human genomes

###### *Arabidopsis thaliana*

busco --cpu 8 -f --mode genome --lineage_dataset brassicales_odb10 --offline --in ${input} --out ${input}.busco --out_path busco

###### *Drosophila melanogaster*

busco --cpu 8 -f --mode genome --lineage_dataset diptera_odb10 --offline --in ${input} --out ${input}.busco --out_path busco

###### *Oryza sativa*

busco --cpu 8 -f --mode genome --lineage_dataset poales_odb10 --offline --in ${input} --out ${input}.busco --out_path busco

###### *Zea mays*

busco --cpu 8 -f --mode genome --lineage_dataset poales_odb10 --offline --in ${input} --out ${input}.busco --out_path busco

#### Asmgene for human genomes

**Map the cDNAs to the reference or assemblies:**

minimap2 -cxsplice:hq -t 10 sample.polish.fasta Homo_sapiens.GRCh38.cdna.all.fa.gz > sample.polish.paf

**Run asmgene:**

paftools.js asmgene -i .99 ref.fa.paf *polish.paf > stat.polish.xls

#### Gene annotation for 35 human genomes using LiftOff

liftoff -p 10 -flank 0.1 -sc 0.85 -copies -g GRCh38.gencode.v35.gff3 -o sample.liftoff.GRCh38.gff3 -u sample.liftoff.GRCh38.unmapped.gene.list -dir sample_LiftOff sample.fa GRCh38.fa

#### Repeat annotation for 35 human genomes using RepeatMasker

RepeatMasker -parallel 20 -engine rmblast -species human -html -gff -s -xsmall -dir sample_RM sample.fa

#### SDs calling using BISER

biser -o sample_Biser -t 20 sample.fa
