# Supplementary material for: NextDenovo: an efficient error correction and accurate assembly tool for noisy long reads
Source: Genome Biol. 2024 Apr 26;25:107. doi: 10.1186/s13059-024-03252-4 (PMC11046930; doi:10.1186/s13059-024-03252-4)
Supplement: Supplementary file 4 — Additional file 4. Review history. [file 13059_2024_3252_MOESM4_ESM.docx]

**1^st^ round**

**Reviewer 1**

The manuscript introduces NextDenovo, a cutting-edge assembler that has been designed to correct long noisy reads, particularly ONT. It employs an attentive approach to repeated regions and adheres to a classical overlap graph for the assembly of reads. Notably, NextDenovo's correction module was evaluated against NECAT and CANU, and the entire assembly procedure was tested against commonly utilized long-read assemblers. NextDenovo demonstrates commendable performance, matching NECAT in both qualitative outcomes and computational efficiency, and notably reducing misassemblies. Furthermore, the tool is adeptly developed, featuring clear instructions, guides, and tutorials to assist end users.

While the manuscript is largely well-structured and clear, certain areas could be further enhanced:

Methodological Section: It would benefit from a more pedagogical treatment to elucidate the complex processes for a broader audience, making the novel techniques more accessible.

Result Presentation: Integrating metadiscourse to delineate raw data descriptions from higher-level insights would add clarity and depth to the results section, fostering a more nuanced understanding.

Major Remarks:

Support for Claims: Several important and non-trivial claims within the manuscript lack supporting data or references. For example:

"However, in terms of the assembly of segmental duplications/repeats, and especially for large plant genome assemblies, the CTA-based strategy usually has an enhanced ability to distinguish different gene copies and produce more accurate and continuous assemblies" - The inclusion of relevant references would validate this assertion.

"This implies that the correction speed advantage of NextDenovo will become increasingly obvious as the read length increases" - This claim should be underpinned by concrete experimental evidence.

*Regarding the impact of error correction on repeat distinction and continuity in NextDenovo assemblies, it might be instructive to conduct an experiment using reads corrected by NextDenovo with other assemblers. This could help to elucidate whether the error correction step or the assembly step is responsible for the observed effect.

Benchmarking of Novel Correction Methods: The manuscript emphasizes innovative correction methods but does not benchmark them against existing tools like Racon, Daccord, CONSENT, or NextPolish. Comparative analysis against these would offer a more comprehensive evaluation.

Minor Remarks:

Table Readability: Highlighting the best results in all tables would enhance reader comprehension.

Ambiguous Sentence: The sentence "Because a large number of noisy or incorrect overlap alignments exist in regions with high error rates, these regions tend to be located within repeat regions." needs to be rephrased to avoid potential misunderstandings.

Terminology: Definitions for specialized terms such as "Dovetail alignment" would be beneficial.

Resource Utilization: Details regarding memory usage and CPU time should be included for a more complete assessment.

Choice of Reference in Figure 2: The rationale behind using Flye as the reference in "Fig. 2: De novo assembly of 35 human genomes. (A) Geographical location of the 35 individuals sequenced. (B) Comparison of 35 human assemblies between NextDenovo and Flye" should be explained to provide context.

In summary, the manuscript presents a significant advancement in genome biology with the introduction of NextDenovo. However, refining the pedagogical aspects, reinforcing unsupported claims, and addressing the minor remarks would elevate the manuscript to a more robust scientific contribution.

**Reviewer 2**

Hu et al present a correction-then-assembly tool for Oxford Nanopore reads. The authors show that this approach is dramatically faster than Canu and 1.1-1.6 times faster than Necat and suggest that with increasing read length speed advantages will increase. In terms of how much data is corrected, the algorithm performs highly similar compared to Canu and Necat (between 1% less and 4.5% more data corrected). The error rate of the corrected reads is 1-2% lower than for Canu and 0-0.4% lower than for Necat with an overall error rate of less than 1%. Using their algorithm on 35 human genomes, the authors identify a number of SDs as well as a gene duplication of AMY1 in a number of individuals.

Overall, faster, and more accurate software is always welcome. However, some doubts remain (at this point) as to whether all assertions in the manuscript are correct, which will require further analysis. Whether this software is likely to be used widely depends in part on more thorough validations that performed so far. As I am traveling, I could unfortunately not install the software. I hope other reviewers will test this.

Major comments:

- page 5, lines 9-10: the corrected data size is very close to what published software achieves (between 1% less and 4.5% more data corrected). This suggests gains without loss of achieved correction, but whether there is data that is not corrected by NextDenovo (but that is corrected by Canu/Necat) is not shown.

- page 5 "resulting in the reads corrected by NextDenovo having a longer average length (90.98 kb) than those
corrected by Canu (84.27 kb) and Necat (89.17 kb)." These data should be shown as distributions, so that the reader can assess if this a uniform shift or caused by few very long corrected reads that are not corrected by the competing algorithms.

- page 5 "NextDenovo can detect these chimeric reads and can split them at the LSRs; 89.10% of the corrected reads can be mapped to reference with >=99% coverage". The authors should investigate how precise this procedure is. How many reads are split that are not really chimeric reads?

- page5: The section "Benchmarking the error correction module" compares to Canu and Necat, but the following section also uses Flye (v2.8) and Wtdbg2 (v2.5). It seems worthwhile to include Flye and Wtdbg2 in the previous section as well.

- for all increases in performance, it is not clear which step in the algorithm causes the improvement. The authors should replace some of their procedures (one at a time) with a simpler published procedure to define the impact of the procedure in question.

- page 7: concerning human segmental duplications: Such SDs are of high interest but given that these are newly called SDs they might also be false positives. The authors should run other pieces of software and then validate experimentally a few SDs that can only be called with their software.

- page 7: the same thought as previously applies to the calling of duplicated genes. The authors should validate the AMY1 duplication with an orthogonal method.

- Methods: "The samples and genome sequence data were not transferred or released outside of China." The authors should apply their algorithm on datasets that are available to all researchers and reevaluate whether SDs and gene duplications can be found.

- what fraction of genes are completely assembled and how does this compare to other pieces of software?

- last but not least, the authors should apply their algorithm to PacBio data (ideally of a similar sample as for Nanopore) and assess problems that arise specifically with one of the machines.


minor comments:

- page6: On average, NextDenovo and Flye produced similar assembly sizes (2.83 Gb) with about 90.84% genome coverage". It seems unlikely that both NextDenovo and Flye have the exact same numbers. Please provide numbers for both as well as for the other pieces of software used.

- The manuscript would benefit from careful proofreading by a native speaker.

- Figure 3 is difficult to decipher

**Authors’ response**

Reviewer 1

The manuscript introduces NextDenovo, a cutting-edge assembler that has been designed to correct long noisy reads, particularly ONT. It employs an attentive approach to repeated regions and adheres to a classical overlap graph for the assembly of reads. Notably, NextDenovo's correction module was evaluated against NECAT and CANU, and the entire assembly procedure was tested against commonly utilized long-read assemblers. NextDenovo demonstrates commendable performance, matching NECAT in both qualitative outcomes and computational efficiency, and notably reducing misassemblies. Furthermore, the tool is adeptly developed, featuring clear instructions, guides, and tutorials to assist end users.

While the manuscript is largely well-structured and clear, certain areas could be further enhanced:

Methodological Section: It would benefit from a more pedagogical treatment to elucidate the complex processes for a broader audience, making the novel techniques more accessible.

Response: Thanks for the suggestion. We have added a general introduction to the NextDenovo algorithm in the Method section to help readers quickly understand the overall algorithmic framework of NextDenovo, “NextDenovo consists of five main steps. The initial step involves pairwise raw read overlapping, followed by the second step which filters the overlapping results to avoid erroneous alignments that affect the error correction accuracy. The third step focuses on error correction based on the filtered overlapping results, while the fourth step entails a two-step iterative pairwise corrected reads overlapping. The final step involves constructing an assembly graph using the overlapping results, followed by graph cleaning and result outputting.”. Then, in the Methods section, we also show a detailed introduction to each step of the NextDenovo algorithm, which can help readers understand the specific details of its algorithm. (Lines 352-360)

Result Presentation: Integrating metadiscourse to delineate raw data descriptions from higher-level insights would add clarity and depth to the results section, fostering a more nuanced understanding.

Response: The results parts have been revised and polished by David Copper, one of the authors of this work. Note: the revised words, grammar or other minor changes that do not modify the meaning of the manuscript are not highlighted in the submitted version.

Major Remarks:

Support for Claims: Several important and non-trivial claims within the manuscript lack supporting data or references. For example:

"However, in terms of the assembly of segmental duplications/repeats, and especially for large plant genome assemblies, the CTA-based strategy usually has an enhanced ability to distinguish different gene copies and produce more accurate and continuous assemblies" - The inclusion of relevant references would validate this assertion.

Response: Thanks for the suggestion. We have added three references:

https://www.ncbi.nlm.nih.gov/pmc/articles/PMC6585154/,

https://pubmed.ncbi.nlm.nih.gov/28298431/,

https://pubmed.ncbi.nlm.nih.gov/33397900/. Moreover, this statement was also verified by the subsequent hybrid strategies that combined the error correction step of other tools with the assembly steps using the ATC-based tools Wtdbg2 and Flye (both tools accept uncorrected long reads as input by default, but can also accept error-corrected reads as input). The result showed that Wtdbg2 and Flye generally produce more contiguous assemblies using corrected reads than using raw data by default. (Lines 76-77)

"This implies that the correction speed advantage of NextDenovo will become increasingly obvious as the read length increases" - This claim should be underpinned by concrete experimental evidence.

Response: Thanks for the suggestion. We have tested simulated reads of varying length and corrected them using NextDenovo, Canu and Necat (we failed to run Consent on these datasets, because it invariably reported a segmentation fault). We found that all software took longer to run as the read length increased. However, with NextDenovo and Necat the time only increased slightly, whereas with Canu it increased significantly. We revised the manuscript accordingly based on this result. (Lines 146-150)

*Regarding the impact of error correction on repeat distinction and continuity in NextDenovo assemblies, it might be instructive to conduct an experiment using reads corrected by NextDenovo with other assemblers. This could help to elucidate whether the error correction step or the assembly step is responsible for the observed effect.

Response: Thank you for a very good suggestion. We have added a paragraph to describe our test results. “To test the performance of different modules of NextDenovo, we used hybrid strategies that combined either the error correction step of NextDenovo with the assembly steps using the ATC-based tools Wtdbg2 and Flye (both tools can accept error-corrected reads as input), or the error correction step of Canu and Necat with the assembly step using NextDenovo. Overall, with a few exceptions, when combined with reads corrected by any tools as input, Wtdbg2 and Flye generally produced more contiguous assemblies than using raw data, and for relatively less complex genomes (A. thaliana and D. melanogaster), NextDenovo and Flye reported similar assemblies, better than Wtdbg2, and for complex genomes (O. sativa and Z. mays), NextDenovo reported much better assemblies than Wtdbg2 and Flye. In addition, Wtdbg2 assemblies using error-corrected reads from NextDenovo were generally more contiguous than those using error-corrected reads from Canu and Necat, with the exception of the O. sativa genome. The Flye assemblies using the error-corrected reads from NextDenovo were more contiguous on the relatively less complex genomes than those using the error-corrected reads from Canu and Necat, but were more fragmented on the complex genomes. When replacing the assembly steps of Canu or Necat with NextDenovo, NextDenovo reported better assemblies than Canu on all test genomes, and NextDenovo produced similar assemblies to Necat on the A. thaliana and O. sativa genomes, better assembly than Necat on the Z. mays genome, but worse assembly than Necat on the D. melanogaster genome (Supplementary Tables 4 and 5).” (Lines 226-243)

Benchmarking of Novel Correction Methods: The manuscript emphasizes innovative correction methods but does not benchmark them against existing tools like Racon, Daccord, CONSENT, or NextPolish. Comparative analysis against these would offer a more comprehensive evaluation.

Response: We have added CONSENT to this benchmark and the results are shown in Table 1. Racon and NextPolish are genome polishing tools (correcting errors in assemblies) and cannot be used to correct errors in sequencing reads. Daccord failed to run, as it requires DALIGNER, and DALIGNER could not perform alignment for NanoPore reads (it requires each read name in fastq files, similar to Pacbio reads). We also tried the newly released error correction tool vechat (https://github.com/HaploKit/vechat), but it did not complete on our test data (only ~12 Gb long reads data) for more than 4 days, so we think that this tool may be unsuitable for performing read error-correction for genome assembly projects. (Lines 137-182, Highlighted parts)

Minor Remarks:

Table Readability: Highlighting the best results in all tables would enhance reader comprehension.

Response: Thanks for the suggestion. We have bolded the best results in all tables.

Ambiguous Sentence: The sentence "Because a large number of noisy or incorrect overlap alignments exist in regions with high error rates, these regions tend to be located within repeat regions." needs to be rephrased to avoid potential misunderstandings.

Response: Thank you for pointing this out. It now reads “Repeated regions typically contain numerous noisy or incorrect overlap alignments. These regions are usually characterized by lower accuracy after the initial correction, …”. (Lines 92-94)

Terminology: Definitions for specialized terms such as "Dovetail alignment" would be beneficial.

Response: Thanks for the suggestion. We have added detailed explanations for some specialized terms in the manuscript. (Lines 111, 116-118, 136-137, 180-182)

Resource Utilization: Details regarding memory usage and CPU time should be included for a more complete assessment.

Response: We’re sorry that we only recorded wall clock time in the previous run, so we cannot give detailed memory usage and CPU time. However, we did record detailed memory usage and CPU time in the newly added test, and these results are given in Supplementary Tables 2 and 11.

Choice of Reference in Figure 2: The rationale behind using Flye as the reference in "Fig. 2: De novo assembly of 35 human genomes. (A) Geographical location of the 35 individuals sequenced. (B) Comparison of 35 human assemblies between NextDenovo and Flye" should be explained to provide context.

Response: We added an explanation “Given Flye performed well in both simulated and real non-human data, we evaluated the performance of NextDenovo and Flye, representing two assembly strategies (CTA and ATC), for human genome assembly (Fig. 2B, Supplementary Table 7).”. (Lines 262-264)

In summary, the manuscript presents a significant advancement in genome biology with the introduction of NextDenovo. However, refining the pedagogical aspects, reinforcing unsupported claims, and addressing the minor remarks would elevate the manuscript to a more robust scientific contribution.

Response: We thank the reviewer for these positive comments. We have made substantial improvements to our manuscript, and hope these changes will fix these issues.

Reviewer 2

Hu et al present a correction-then-assembly tool for Oxford Nanopore reads. The authors show that this approach is dramatically faster than Canu and 1.1- 1.6 times faster than Necat and suggest that with increasing read length speed advantages will increase. In terms of how much data is corrected, the algorithm performs highly similar compared to Canu and Necat (between 1% less and 4.5% more data corrected). The error rate of the corrected reads is 1-2% lower than for Canu and 0-0.4% lower than for Necat with an overall error rate of less than 1%. Using their algorithm on 35 human genomes, the authors identify a number of SDs as well as a gene duplication of AMY1 in a number of individuals.

Overall, faster, and more accurate software is always welcome. However, some doubts remain (at this point) as to whether all assertions in the manuscript are correct, which will require further analysis. Whether this software is likely to be used widely depends in part on more thorough validations that performed so far. As I am traveling, I could unfortunately not install the software. I hope other reviewers will test this.

Major comments:

- page 5, lines 9-10: the corrected data size is very close to what published software achieves (between 1% less and 4.5% more data corrected). This suggests gains without loss of achieved correction, but whether there is data that is not corrected by NextDenovo (but that is corrected by Canu/Necat) is not shown.

Response: Thanks for the suggestion. We have analyzed this case and described it as “Further examination unveiled that about 0.93% of simulated reads and 2.36% of real biological reads could be corrected by either Canu or Consent, but not by NextDenovo (Supplementary Fig. 1). It is imperative to highlight that within this subset of uncorrected reads, 99.98% of simulated reads and 99.08% of real biological reads cannot be completely mapped to the reference genome (80% coverage), this indicates that the majority of these uncorrected reads were of extremely low quality or chimeric and were filtered out by NextDenovo to prevent any adverse effects on the subsequent assembly graph construction.”. The Necat correction reads have been renamed, so they cannot be used for this comparison. (Lines 153-159) - page 5 "resulting in the reads corrected by NextDenovo having a longer average length (90.98 kb) than those corrected by Canu (84.27 kb) and Necat (89.17 kb)."

These data should be shown as distributions, so that the reader can assess if this a uniform shift or caused by few very long corrected reads that are not corrected by the competing algorithms.

Response: Thank you for pointing this out. We performed some additional testing and found that NextDenovo could not always maintain this advantage, so we deleted this statement from the manuscript. (Line 173)

- page 5 "NextDenovo can detect these chimeric reads and can split them at the LSRs; 89.10% of the corrected reads can be mapped to reference with >=99% coverage".

The authors should investigate how precise this procedure is. How many reads are split that are not really chimeric reads?

Response: Thanks for the suggestion. We analyzed the accuracy of this procedure and described the result as “We also investigated how accurate this chimeric read splitting process is. The results showed that only 0.07% of reads were split by error, lower than the 4.83% for Canu but higher than the 0.002% for Consent (a mis-split read was defined as a read that can be completely mapped to the reference without correction, but was not included in the corrected result or the length was significantly shorter after correction)”. The Necat correction reads have been renamed, so they could not be used for this analysis. (Lines 178-182)

- page5: The section "Benchmarking the error correction module" compares to Canu and Necat, but the following section also uses Flye (v2.8) and Wtdbg2 (v2.5). It seems worthwhile to include Flye and Wtdbg2 in the previous section as well.

Response: Flye and Wtdbg2 are ATC-based assemblers which do not have a long- read error-correction module. However, we have added CONSENT (a tool that focuses on the long-read error-correction and assembly polishing) to this benchmark and the results are shown in Table 1. (Lines 137-182, Highlighted parts)

- for all increases in performance, it is not clear which step in the algorithm causes the improvement. The authors should replace some of their procedures (one at a time) with a simpler published procedure to define the impact of the procedure in question.

Response: Thank you for a very good suggestion. We have added a paragraph to describe our test results. “To test the performance of different modules of NextDenovo, we used hybrid strategies that combined either the error correction step of NextDenovo with the assembly steps using the ATC-based tools Wtdbg2 and Flye (both tools can accept error-corrected reads as input), or the error correction step of Canu and Necat with the assembly step using NextDenovo. Overall, with a few exceptions, when combined with reads corrected by any tools as input, Wtdbg2 and Flye generally produced more contiguous assemblies than using raw data, and for relatively less complex genomes (A. thaliana and D. melanogaster), NextDenovo and Flye reported similar assemblies, better than Wtdbg2, and for complex genomes (O. sativa and Z. mays), NextDenovo reported much better assemblies than Wtdbg2 and Flye. In addition, Wtdbg2 assemblies using error-corrected reads from NextDenovo were generally more contiguous than those using error-corrected reads from Canu and Necat, with the exception of the O. sativa genome. The Flye assemblies using the error-corrected reads from NextDenovo were more contiguous on the relatively less complex genomes than those using the error-corrected reads from Canu and Necat, but were more fragmented on the complex genomes. When replacing the assembly steps of Canu or Necat with NextDenovo, NextDenovo reported better assemblies than Canu on all test genomes, and NextDenovo produced similar assemblies to Necat on the A. thaliana and O. sativa genomes, better assembly than Necat on the Z. mays genome, but worse assembly than Necat on the D. melanogaster genome (Supplementary Tables 4 and 5).” (Lines 226-243)

- page 7: concerning human segmental duplications: Such SDs are of high interest but given that these are newly called SDs they might also be false positives. The authors should run other pieces of software and then validate experimentally a few SDs that can only be called with their software.

Response: We do not consider it necessary to run other software to reduce the false positive rate. Firstly, the BISER exhibited high performance on simulated data (99.9% detection rate even for high error rate genome, https://doi.org/10.1186/s13015-022- 00210-2). It has also been applied to the Human T2T-CHM13 genome (https://doi.org/10.1126/science.abj6965). Secondly, Ko et al. revealed that 4~16% of duplication regions are falsely assembled based on the genomes of the Vertebrate Genomes Project. Moreover, the main source of false duplication callings is the mis- assembly of paralogs from the two divergent haplotype sequences of the diploid sample (https://genomebiology.biomedcentral.com/articles/10.1186/s13059-022- 02764-1, https://www.nature.com/articles/s41586-021-03451-0). Therefore, we think the number of false SDs is limited. This notwithstanding, larger and highly similar SDs cannot be assembled by high error-rate long reads. Only 32% of SDs are resolved in the genome assembled by Pacbio CLR reads (https://onlinelibrary.wiley.com/doi/epdf/10.1111/ahg.12364). That is why most AMY copies cannot be resolved by Nanopore long reads (below results). All the blood samples were used for sequencing, leaving no surplus samples for experimental validation of SDs.

- page 7: the same thought as previously applies to the calling of duplicated genes. The authors should validate the AMY1 duplication with an orthogonal method.

Response: We validated the AMY1 duplication with a read-depth method. In short, we mapped the raw long reads to the Human T2T-CHM13 genome, and calculated the read depth of AMY genes. We can estimate the copy number of each AMY gene by a simple comparison of the coverage within the AMY gene and its flanking regions. Although most samples contain multiple copies of the AMY gene, based on the coverage of AMY genes (below picture), NextDenovo can assemble some copies of AMY (Sample name: A02, A03, A06, Iran, Y1, Y10, Y4, Y5, Y6, Y7) whereas Flye only assembles extra copies of AMY1C gene in sample Y3.

(Insert Figure Here)

Figure: Depth of the AMY gene cluster across 35 human genomes. The red boxes denote the locations of the five AMY genes. The red texts indicate the AMY genes with extra copy numbers assembled by NextDenovo.

- Methods: "The samples and genome sequence data were not transferred or released outside of China." The authors should apply their algorithm on datasets that are available to all researchers and reevaluate whether SDs and gene duplications can be found.

Response: Sorry for the confusion. According to Chinese government rules, we uploaded all raw reads sequencing data and genome assemblies of 35 individuals to GSA-Human (https://ngdc.cncb.ac.cn/gsa-human) for controlled-access purposes. The data can only be downloaded after being authorized by its Data Access Committee. Once authorized, the data can be transferred or shared outside of China. Please refer to the GSA-Human Request Guide for Users (https://ngdc.cncb.ac.cn/gsa- human/document/GSAHuman_Request_Guide_for_Users_us.pdf). Additionally, we have already shared all SD and gene duplication results on Zenodo(https://zenodo.org/record/8418409).

- what fraction of genes are completely assembled and how does this compare to other pieces of software?

Response: We have evaluated the integrity of the genes. The non-human assembly evaluation results are shown in Table 2 and Supplementary Table 3 and the 35 human assembly evaluation results are shown in Supplementary Tables 7 and 8.

- last but not least, the authors should apply their algorithm to PacBio data (ideally of a similar sample as for Nanopore) and assess problems that arise specifically with one of the machines.

Response: Thanks for the suggestion. We have evaluated the performance of NextDenovo on PacBio HiFi data (the only data currently generated by PacBio) and compared it to Hifiasm (an assembly tool developed specifically for HiFi data). We found that NextDenovo can be used for HiFi data assembly, but its assembly quality is significantly lower than Hifiasm. We now point this out in the manuscript. (Lines 333- 335)

minor comments:

- page6: On average, NextDenovo and Flye produced similar assembly sizes (2.83 Gb) with about 90.84% genome coverage". It seems unlikely that both NextDenovo and Flye have the exact same numbers. Please provide numbers for both as well as for the other pieces of software used.

Response: Thanks for the suggestion. We have listed all detailed values in the Supplementary Tables 7. We have also deposited all human assemblies at the Genome Sequence Archive (https://ngdc.cncb.ac.cn/gsa-human/) at the National Genomics Data Center, China National Center (NGDC) for Bioinformation/Beijing Institute of Genomics, Chinese Academy of Sciences, under accession number PRJCA006287.

- The manuscript would benefit from careful proofreading by a native speaker.

Response: The manuscript has been revised by David Copper, one of the authors of this work. Note: the revised words, grammar or other changes that do not modify the meaning of the manuscript are not highlighted in the submitted version.

- Figure 3 is difficult to decipher

Response: We already update all main figures to the vector graphic

**2^nd^ round**

**Reviewer 1**

I am pleased with the authors' thorough response to the revision suggestions. They have enhanced the manuscript significantly by clarifying the methodological section, making complex processes more understandable. The results section is improved for better clarity, and important claims are now well-supported with relevant data and references. The comparison of novel correction methods against existing tools provides a comprehensive evaluation of NextDenovo's performance. Additionally, the authors have efficiently addressed minor remarks, improving readability and detail. Overall, their efforts have elevated the manuscript, making it a robust and valuable contribution to genome biology.

**Reviewer 2**

Hu et al have significantly revised their paper and present answers to most of the questions I raised. Broadly speaking, these answers are satisfactory.
Unfortunately, the authors have not taken up my question to define SDs that can only be found with their software - and then to validate them experimentally (using non-computational methods). Thus, we don't fully know if their algorithm brings truly novel and trustworthy SDs to the table. That is a shortcoming, but on the other hand, the software does seem to offer good performance overall and is therefore likely to find a user base.
